# Prevalence and risk factors for antibiotic utilization in Chinese children

**DOI:** 10.1186/s12887-021-02706-z

**Published:** 2021-06-01

**Authors:** Shasha Guo, Qiang Sun, Xinyang Zhao, Liyan Shen, Xuemei Zhen

**Affiliations:** 1 Centre for Health Management and Policy Research, School of Public Health, Cheeloo College of Medicine, Shandong University, Jinan, 250012 China; 2 NHC Key Lab of Health Economics and Policy Research (Shandong University), Jinan, 250012 China; 3 School of Nursing, China Medical University, Shenyang, 110100 China

**Keywords:** Antibiotic, Prevalence, Risk factors, Children, China

## Abstract

**Background:**

Antibiotic resistance poses a significant threat to public health globally. Irrational utilization of antibiotics being one of the main reasons of antibiotic resistant. Children as a special group, there's more chance of getting infected. Although most of the infection is viral in etiology, antibiotics still are the most frequently prescribed medications for children. Therefore, high use of antibiotics among children raises concern about the appropriateness of antibiotic prescribing. This systematic review aims to measuring prevalence and risk factors for antibiotic utilization in children in China.

**Methods:**

English and Chinese databases were searched to identify relevant studies evaluating the prevalence and risk factors for antibiotic utilization in Chinese children (0-18 years), which were published between 2010 and July 2020. A Meta-analysis of prevalence was performed using random effect model. The Agency for Healthcare Research and Quality (AHRQ) and modified Jadad score was used to assess risk of bias of studies. In addition, we explored the risk factors of antibiotic utilization in Chinese children using qualitative analysis.

**Results:**

Of 10,075 studies identified, 98 eligible studies were included after excluded duplicated studies. A total of 79 studies reported prevalence and 42 studies reported risk factors for antibiotic utilization in children. The overall prevalence of antibiotic utilization among outpatients and inpatients were 63.8% (35 studies, 95% confidence interval (*CI*): 55.1-72.4%), and 81.3% (41 studies, 95% *CI*: 77.3-85.2%), respectively. In addition, the overall prevalence of caregiver’s self-medicating of antibiotics for children at home was 37.8% (4 studies, 95% *CI*: 7.9-67.6%). The high prevalence of antibiotics was associated with multiple factors, while lacking of skills and knowledge in both physicians and caregivers was the most recognized risk factor, caregivers put pressure on physicians to get antibiotics and self-medicating with antibiotics at home for children also were the main factors attributed to this issue.

**Conclusion:**

The prevalence of antibiotic utilization in Chinese children is heavy both in hospitals and home. It is important for government to develop more effective strategies to improve the irrational use of antibiotic, especially in rural setting.

**Supplementary Information:**

The online version contains supplementary material available at 10.1186/s12887-021-02706-z.

## Background

Antibiotic utilization is a major driver of antibiotic resistance, which is becoming a serious public health threat, imposing a direct effect on morbidity, mortality, and financial burden [1–3]. It was reported that China was the second largest consumer of antibiotics, and up to 57% of the increase in antibiotic consumption in the hospital sector among BRICS countries (Brazil, Russia, India, China, and South Africa) was attributable to China [4]. In addition, China also has the most rapid growth rate of antibiotic resistance worldwide [5].

Antibiotics are the most widely prescribed therapy among all medications given to children [6, 7]. It was reported that the proportion of children with antibiotic prescription in the hospital settings was between 22% and 78 %[8–13], at the same time, inappropriate antibiotic prescription was the most common in pediatric clinical practice [14, 15]. About 50% of antibiotics were prescribed to children who were suffering from viral infection or non-infectious diseases, and the proportion of antibiotic prescriptions under 15 years old was three times than other ages [16, 17]. In addition, antibiotic abuse and misuse can also result in adverse events and drug toxicity due to children’s special physiological condition [18]. Children who prescribed antibiotics were more likely to have a subsequent acute bronchitis episode [19, 20]. China Antimicrobial Resistance Surveillance System (CARSS) showed that the proportions of gram-positive bacteria isolated from children and newborns was 45.5% in 2017, which were higher than that of the adults [21]. Children may have little or even no benefit from antibiotics [22], therefore, promotion of rational use of antibiotics and reduction in antibiotic resistance in children has become an urgent problem [23, 24]. To combat this problem, the Chinese government has launched relevant policies continuously since 2009. In 2009, to improve rational use of medicines, China government enacted the national essential medicines scheme, which aimed to curb the use of antibiotics by breaking the financial relation between drug prescription and physician income. Zero-mark-up policy was introduced to primary healthcare centers (PHCs) firstly in 2009, then followed by secondary hospitals in 2012, and expanded in tertiary hospitals in 2016 [25]. Hierarchical management was carried out for the clinical use of antibacterial drugs, which includes three levels: unrestricted use, restricted use and special use, according to safety, effect, drug resistance, and price in 2012 [26]. Since 2013, to improve antibiotic utilization in public hospitals, National Antibiotic Stewardship Program (NASP) was implemented nationwide which limits the proportion of outpatient antibiotic prescriptions and inpatient antibiotic prescriptions in hospitals to 20% and 60%, respectively [27]. National action plan combating antimicrobial resistance proposed to carry out health education on the rational use of antibiotics in primary and secondary schools [28].

Recently, the number of studies on the prevalence of antibiotic utilization in Chinese children has increased greatly, while sparse studies explored risk factors. Assessment of risk factors for antibiotic utilization in children may lead to a better understanding of the prevalence rates of antibiotics utilization and therefore leading to a more effective preventive strategy. In addition, most of the relevant studies were published in Chinese, with only a few in English language, which caused a lack of awareness of antibiotic utilization in China worldwide. There has not been an in-depth, up-to-date, and comparative analysis of the contemporary literature reporting the prevalence and risk factors for antibiotic utilization in Chinese children. In this study, we aimed to summarize the proportion of antibiotic usage among outpatients, inpatients, and self-medication, and investigate the major risk factors for antibiotic utilization in Chinese children.

## Methods

This systematic review and meta-analysis was conducted in accordance with the Preferred Reporting Items for Systematic Reviews and Meta-Analyses (PRISMA) reporting guideline [29]. The protocol was registered with PROSPERO (CRD42020200172) (Table S1 in Additional file 1).

### Search strategy

We searched three Chinese databases including China National Knowledge Infrastructure (CNKI), Chongqing VIP, and Wanfang Data, and three English databases including PubMed, Web of Science, and Embase between January 1, 2010 and July 10, 2020. Search terms were a combination of antibiotic, children, China, and prevalence (or risk factor). Then, references lists of the included studies were screened to complement our database searches. The detailed search strategies were presented in Table S2 Additional file 1.

### Inclusion and exclusion criteria

Inclusion criteria were: (1) studies published in English or Chinese language; (2) publication date was between January 1, 2010 and July 10, 2020; (3) original studies using any study designs, such as observational studies (cohort, case-control, cross-sectional) or randomized studies; (4) reports on children (≤18 years old); (5) reports in China; (6) reports on prevalence rates or risk factors for antibiotic utilization. In order to update the analysis reflecting current patterns and practice guidelines, we excluded studies published before 2010. Children with special diseases (e.g. cancer, leukemia) were excluded as well. For randomized studies, we only included data of baseline or control group. Two reviewers (XY and GS) independently estimated the titles and abstracts of the retrieved studies using the above eligibility criteria, then, full texts were examined. Any discrepancies were resolved by the third reviewer (XM).

### Data extraction

A pre-designed standardized extraction form was used to record the characteristics of each study including first author, publication year, study period, study design, geographical regions, study setting (rural: “rural” refer to place where laborers mainly engaged in agricultural production; urban: “urban” or “city” are cities which are officially administered at the national level as cities [30]), hospital levels (Level 1 hospital: primary hospitals or health institutions offer preventive, clinical treatment, health care and rehabilitation service in community. Generally, a primary hospital has 20-99 ward beds; Level 2 hospital: regional hospitals offer comprehensive medical and health services to multiple communities and offer medical training and research. Ward beds of Level 2 hospitals are between 100 and 499; Level 3 hospital: tertiary hospitals serve multiple regions, offer high-level and specialized medical services and are responsible for higher education and specific research. Each Level 3 hospital has over 500 ward beds [31]), sample size (number of children), age, number of children with antibiotics, number of children with one antibiotic, number of children with antibiotic combination, the top 3 types and utilization rates of antibiotics, and risk factors for antibiotic utilization.

### Statistical analysis

For statistical analysis, STATA version 15.0 was employed. We assessed heterogeneity of prevalence estimates using *I*^2^ index, which was categorized as low (0-25%), moderate (26-50%), and high (above 50% )[32].A random-effect meta-analysis was used to calculate the overall pooled prevalence of antibiotic utilization in children with 95% confidence interval (*CI*) due to high heterogeneity. Subgroup analyses for prevalence were performed with respect to study location (outpatient, inpatient, and self-medication at home), geographical region (eastern, central, and western), setting (urban and rural area), hospital levels (level 1, 2, 3), quantity of antibiotic use (alone or in combination), simple size, study period.

### Study quality assessment

We assessed the included study quality using the Agency for Healthcare Research and Quality (AHRQ) for observational studies [33]. We considered 11-item checklist for quality assessment, including source of investigation, inclusion and exclusion criteria, time period, consecutive of subjects, objectivity of indicators, reproducibility of indicators, reason for subject exclusions, confounding controlled, missing data handled, response rate and completeness of data collection, and results of follow-up. “No” or “Unclear” was scored “0”, and “Yes” was scored “1”. We considered studies that ≤ 3 scores as low quality, 4-7 scores as moderate quality, ≥8 scores as high quality. For randomized studies, we used modified Jadad score with classification criteria of high quality (6-7 scores), moderate quality (4-5 scores), and low quality (≤3), which included the generation of random sequences, randomization, blinding, follow-up. “No” was scored “0”, “unclear” was scored “1”, and “Yes” was scored “2” [34].

## Results

### Study selection

**Fig. 1:**
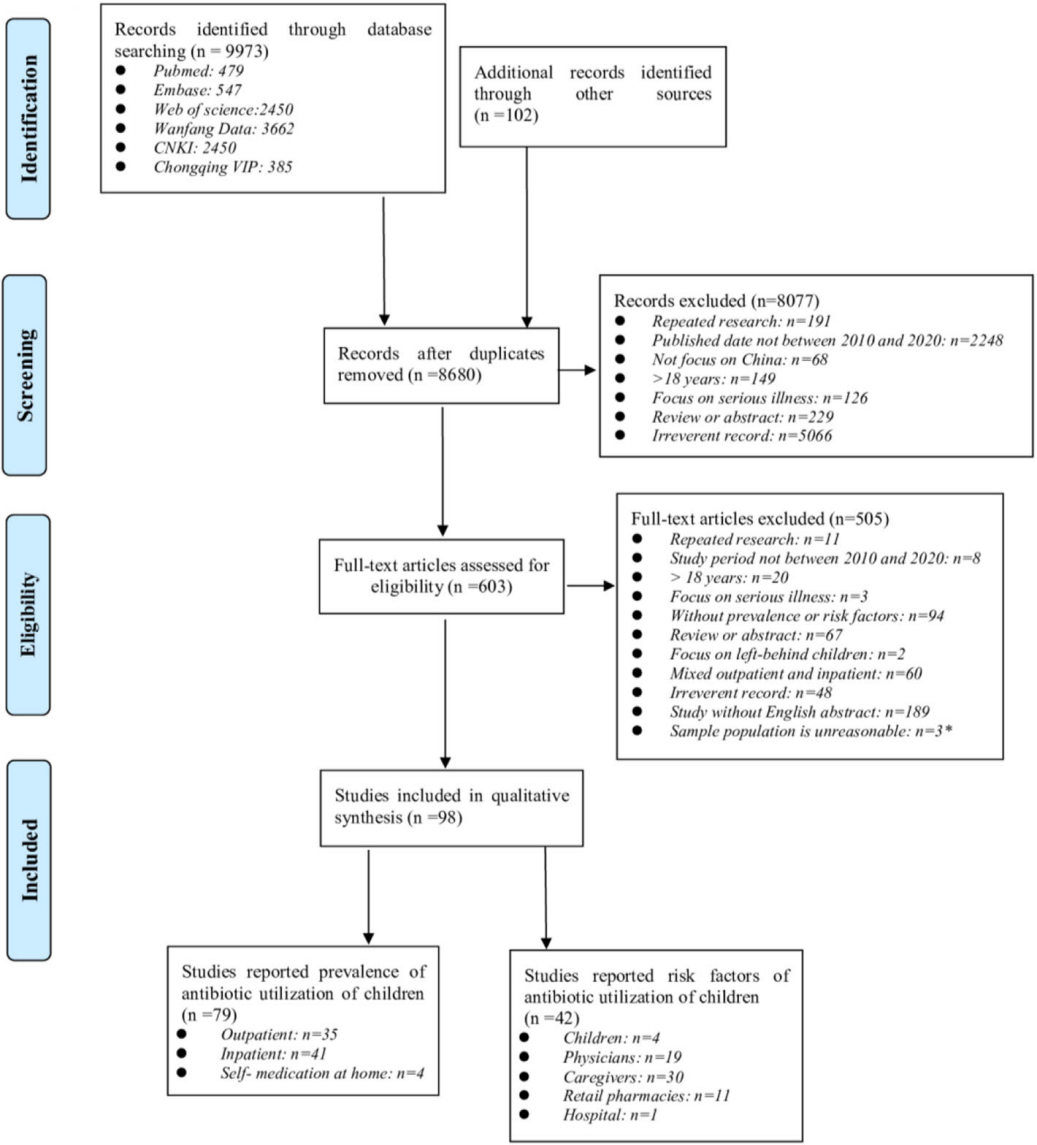
PRISMA flowchart of study selection process. PRISMA: Preferred Reporting Items for Systematic Reviews and Meta-Analyses. CNKI: China National Knowledge Infrastructure. As all subjects in these 3 referenced articles received antibiotics (100% prevalence) as inpatients with bacterial infections, references [158–160] were excluded from the meta-analysis

A total of 9,973 records were retrieved in the initial literature search, and additional 102 studies were identified through manual reference screens. After removing 1,395 duplicates, 8,680 studies were retrieved. Titles and abstracts screening resulting in 603 records for full-text evaluation. Finally, 98 records were included (Fig. 1).

### Study characteristics and quality

Of the 98 eligible studies included in our review, 79 studies reported the prevalence of antibiotic utilization of children, including 34 studies on outpatient [35–68], 40 studies on inpatient [69–108] , and one study on both outpatient and inpatient [109]. Moreover, four studies reported the prevalence of self-medication antibiotic utilization at home [110–113]. The majority of the studies were retrospective observational studies (*n=*73), 6 studies were randomized controlled study, and majority of the studies were conducted in urban area (*n=*72), 7 studies conducted in rural area, and collected data from the Level 3 hospital (*n=*53). The study data were obtained from 22 provinces in mainland China, with the largest number of studies from Guangdong province (*n=*14), followed by Guangxi province (*n=*9), and Jiangsu province (*n=*7). A total of 49 studies collected data from the eastern economic zone, 17 studies from the western economic zone, 10 studies from the central economic zone, and three studies reported data from nationwide. The sample sizes ranged from 45 to 37211. In addition, 42 studies reported the risk factors for antibiotic utilization [20, 35, 36, 42, 45, 46, 52, 53, 58, 60, 67, 70, 76, 77, 83–85, 91, 92, 99, 110–131], of which, four studies focused on children, 19 studies focused on physicians, 30 studies reported from caregivers, 11 studies reported from retail pharmacies, and one study reported from hospital.(Table S3, Table S4, Table S5,Table S6 in Additional file 1).

Regarding the quality of included studies, for observational studies, 36 were with high quality and 56 were with moderate quality (Table S7 in Additional file 1). For randomized studies, 2 were with high quality, 4 were with moderate quality. (Table S8 in Additional file 1).

### Prevalence of antibiotic utilization in Chinese children

**Fig. 2:**
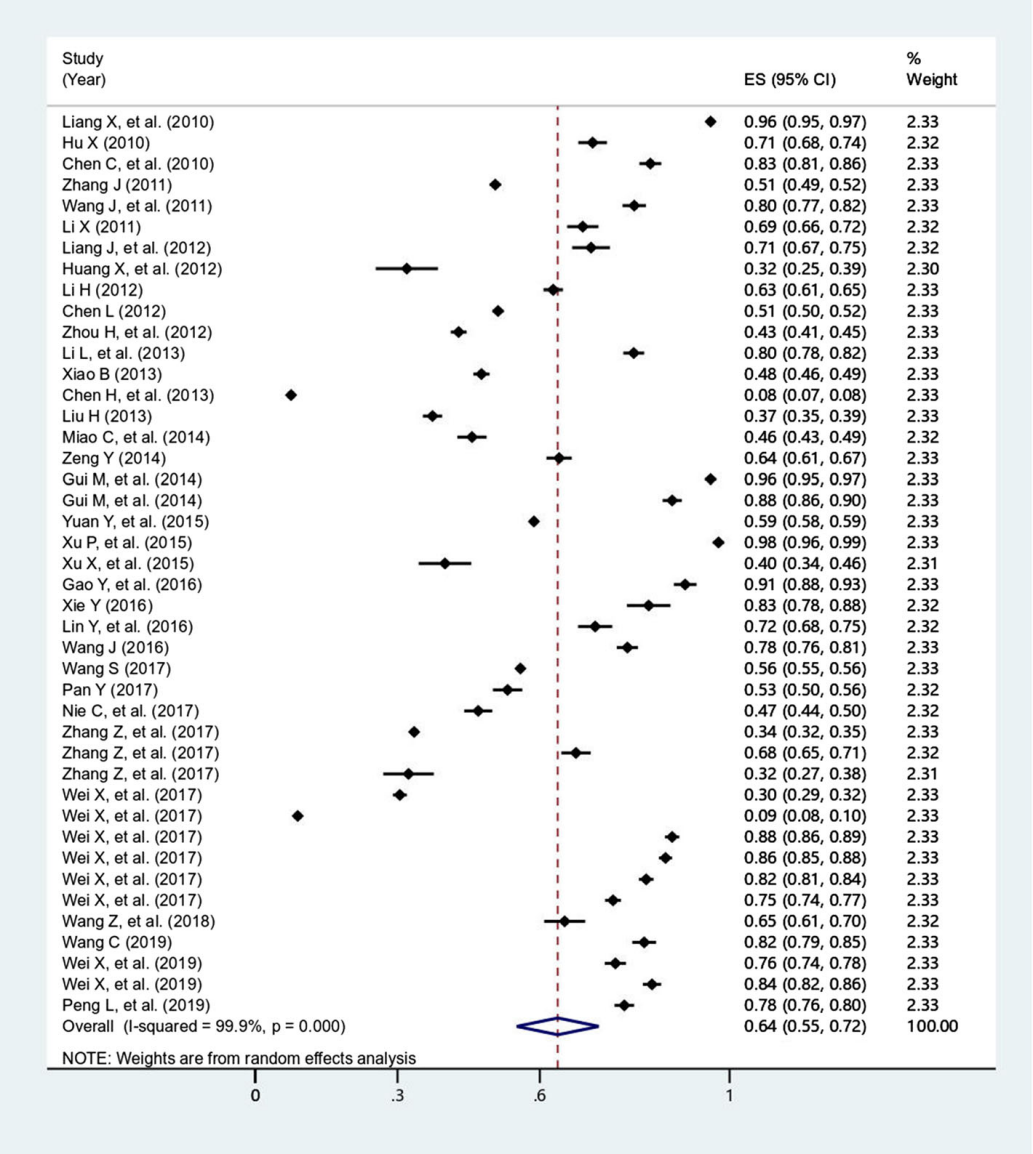
Forest plot of the studies for prevalence of antibiotic utilization of outpatient

**Fig. 3:**
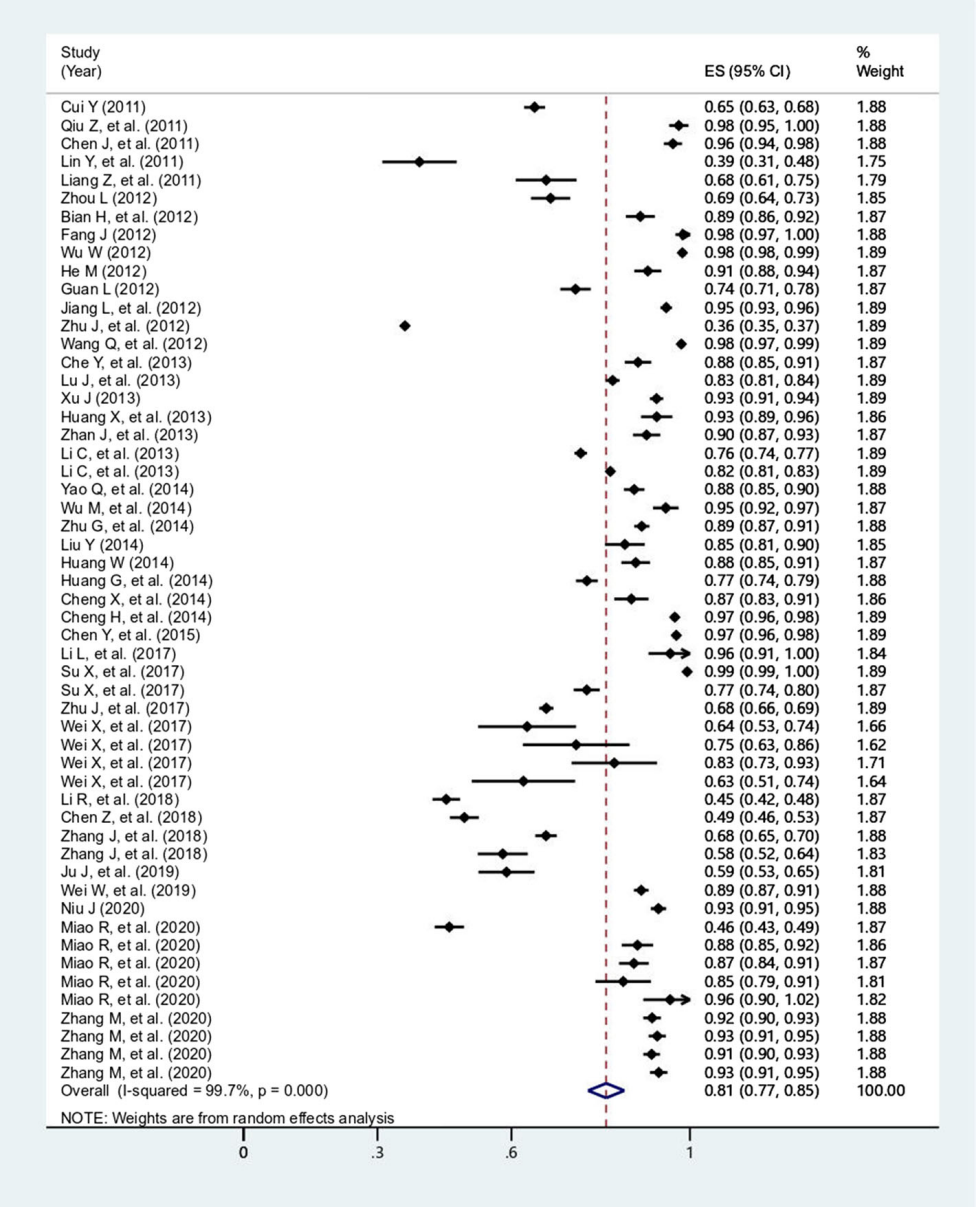
Forest plot of the studies for prevalence of antibiotic utilization of inpatient

**Fig. 4.**
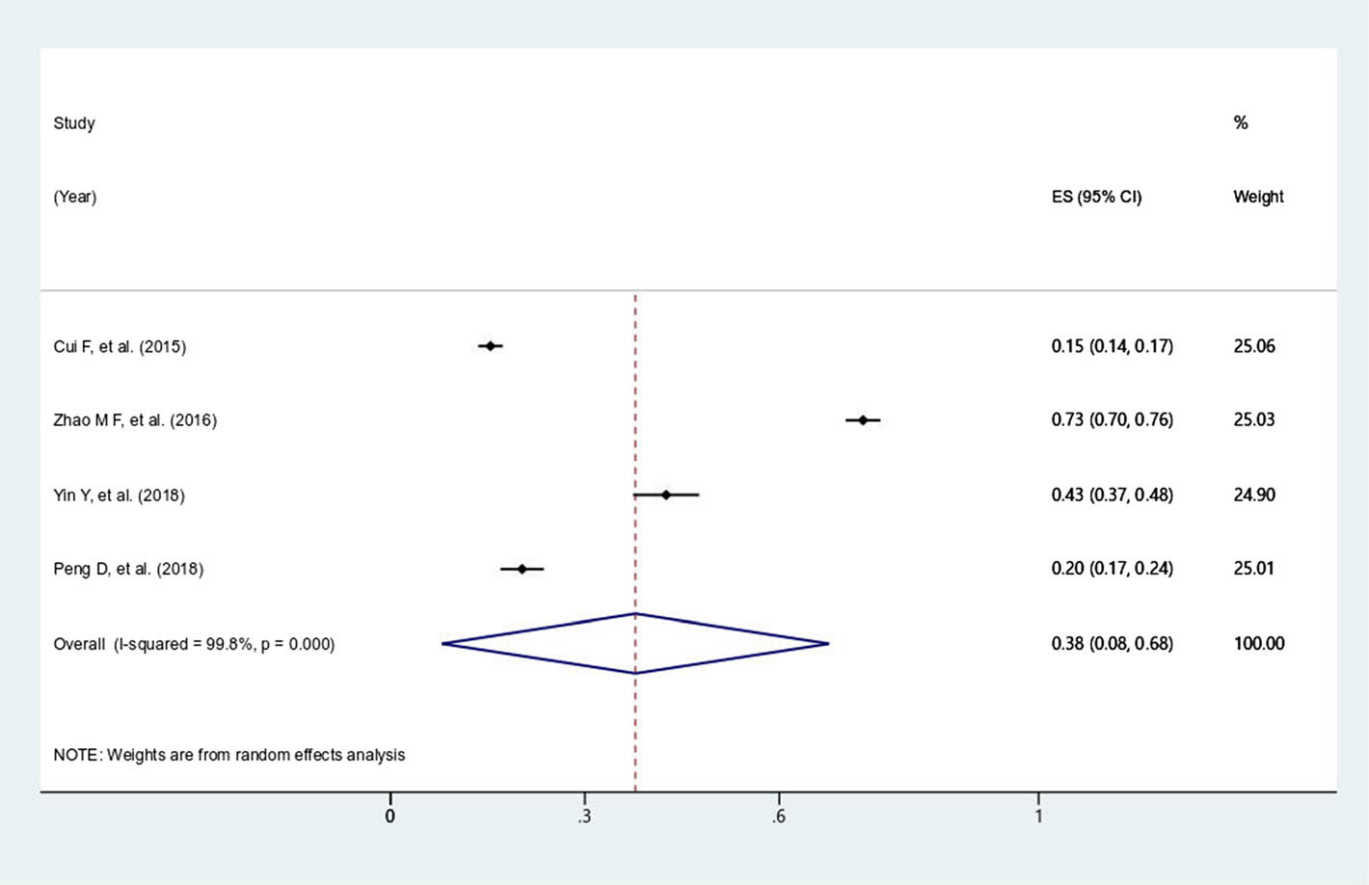
Forest plot of the studies for prevalence of self-medicating at home

The overall prevalence of antibiotic utilization among outpatients and inpatients were 63.8% (95% *CI*: 55.1-72.4%, *I*^*2*^=99.9%, *P*<0.0001) (Fig. 2), and 81.3% (95% *CI*: 77.3-85.2%, *I*^*2*^=99.7%, *P*<0.0001) (Fig. 3), respectively. In addition, the overall prevalence of caregiver’s self-medicating of antibiotics for children at home was 37.8% (95% *CI*: 7.9-67.6%, *I*^*2*^=99.8%, *P*<0.0001) (Fig. 4).

In the subgroup analyses, the prevalence of combined use of antibiotic was 25.2% among outpatients, and 40.2% among inpatients. The prevalence of antibiotic utilization in eastern, central, and western economic zone were 59.8%, 80.0%, 70.0%, respectively, for outpatients, and 81.0%, 78.9%, 80.5%, respectively, for inpatients. A higher prevalence antibiotic utilization was found in urban, with 64.1% and 81.7% for outpatients and inpatients respectively. In addition, prevalence of antibiotic utilization in level 1, 2, 3 hospital were 71.3%, 57.3% 64.0%, respectively, among outpatients, and 85.5% in level 2 hospital, 79.7% in level 3 hospital among inpatients. The percentage of antibiotic utilization fluctuated over time. In outpatient department, it declined between from 2010 to 2013, and in recent years, there has been an upward trend. For inpatient department, it increased from 2010 to 2013, then decrease from 2014-2017, and in recent years, there has been an upward trend. In outpatient department, the prevalence of antibiotic prescription prevalence was 57.7% for studies that included a sample of above 5000, and it was 64.8% for studies that included a sample of 5000 and less than 5000; in inpatient department, the prevalence of antibiotic prescription prevalence was 82.0% for studies that included a sample of above 1000, and it was 81.0% for studies that included a sample of 1000 and less than 1000 (Tables 1 and 2). In addition, we found that the most frequently used antibiotics were the third-generation cephalosporins, penicillins, and macrolides.

**Table 1:** The prevalence of outpatient antibiotic utilization by antibiotic combination situation, economic zone, study setting, and hospital level.

	No. of studies (N)	n/N	Percentage (95% CI) (%)
**Antibiotic combination situation (23)**			
Single use of antibiotic	23	28467/36751	74.8 (68.2-81.3)
Combined use of antibiotic	23	8284/36751	25.2 (18.7-31.8)
**Economic zone (35)**			
Eastern	24	74716/134667	59.8 (49.3-70.2)
Central	4	4363/5244	80.0 (67.2-92.8)
Western	7	5371/8540	70.0 (56.1-83.9)
**Study setting (35)**			
Urban	29	68961/118022	64.1 (54.4-73.8)
Rural	6	15489/30429	63.1 (44.3-82.0)
**Hospital level (35)**			
Level 3	19	61551/103670	64.0 (54.9-73.0)
Level 2	9	14214/33478	57.3 (37.1-77.6)
Level 1	8	8658/11303	71.3 (63.0-79.6)
**Study period (33)**			
2010-2011	15	66803/10992	68.5 (58.5-78.4)
2012-2013	9	10607/22511	54.5 (35.0-74.1)
2014-2015	12	16919/31424	65.2 (49.3-81.1)
2016-2018	7	24033/39501	68.6 (59.0-78.1)
**Sample size (35)**			
≤5000	29	31832/55737	64.8 (53.5-76.1)
>5000	6	52618/92714	57.7 (39.3-76.0)

N: Sample Size; n: Number of Children with Antibiotics; random-effect meta-analysis was used to calculate the overall pooled prevalence of antibiotic utilization. For studies reported different economic zone, study setting, hospital level, study period, sample size, we conducted meta- analysis more than once. Two studies study period was in 2009, therefore, there were 33 studies included subgroup analysis of study period.

**Table 2:** The prevalence of inpatient antibiotic utilization by antibiotic combination situation, economic zone, study setting, and hospital level.

	No. of studies (N)	n/N	Percentage (95% CI) (%)
**Antibiotic combination situation (31)**			
Single use of antibiotic	31	14591/24236	59.8 (51.0-68.6)
Combined use of antibiotic	31	9654/24236	40.2 (31.4-49.0)
**Economic zone (41)**			
Eastern	25	29815/36466	81.0 (77.3-84.7)
Central	9	17707/22043	78.9 (71.9-86.0)
Western	13	23205/32583	80.5 (71.2-89.8)
**Study setting (41)**			
Urban	40	4420/58190	81.7 (77.5-86.0)
Rural	2	219/296	76.3 (62.3-90.3)
**Hospital level (41)**			
Level 3	31	41159/54494	79.7 (74.7-84.6)
Level 2	11	3437/3947	85.5 (81.6-89.5)
Level 1	1	43/45	95.6(-)
**Study period (35)**			
2010-2011	22	30276/40486	82.9 (77.4-88.3)
2012-2013	11	10748/12445	87.9 (84.3-91.4)
2014-2015	8	9232/11193	82.9 (75.8-89.9)
2016-2017	6	5801/ 8209	67.6 (57.1-78.1)
2018-2019	4	3506/4351	82.3 (72.9-91.7)
**Sample size (41)**			
≤1000	27	12534/15777	81.0 (76.8-85.3)
>1000	15	32105/42709	82.0 (73.9-90.2)

N: Sample Size; n: Number of Children with Antibiotics; random-effect meta-analysis was used to calculate the overall pooled prevalence of antibiotic utilization. For studies reported different economic zone, study setting, hospital level, study period, sample size, we conducted meta- analysis more than once. Six studies study period was before 2010, therefore, there were 35 studies included subgroup analysis of study period.

### Risk factors of antibiotic utilization in Chinese children

We explored the risk factors of antibiotic utilization in Chinese children using qualitative analysis from five aspects and 12 items (Table 3). The presentation of factors here is grouped into those at children level (e.g. distribution of disease, lack of skills and knowledge), and physician level (e.g. lack of skills and knowledge, pressure from patient, physician-patient relationship, economic incentive and profit from prescribing medicine, lack of pathogen detection or low pathogen detection rate), and caregiver level (e.g. lack of skills and knowledge, put pressure on physician to get antibiotics, behavior of self-medicating with antibiotics at home for children) and retail pharmacies level (e.g. sale antibiotics without prescription) and hospital level (e.g. ward capacity).

**Table 3:** Risk factors of antibiotic utilization in children in China.

	Risk factors		No. of studies (***N***=42)
**Children**	distribution of disease	The biological systems and organs of children are not well-developed, especially those of younger children, which make children more vulnerable. Children with upper respiratory tract infections (URTIs) are among the highest receivers of antibiotics.	3 (7.1%)
	lack of skills and knowledge	Middle school students still have problems in medication adherence, the management of expired drugs and the antibiotics cognition.	1 (2.4%)
**Physicians**	lack of skills and knowledge	Physicians consider antibiotics to be anti-inflammatory drugs is a common misconception. Doctors might overprescribe antibiotics due to lack of knowledge of its rational use. Gaps between reported knowledge and actual practice within antibiotic prescribing are commonly encountered.	19 (45.2%)
	pressure from patient	Majority of the village doctors would prescribe antibiotics if their patients stick to getting them.	5 (11.9%)
	physician-patient relationship	Ineffective communication between patients and physicians may lead to the unnecessary prescription of antibiotics.	2 (4.8%)
	economic incentive and profit from prescribing medicine	Retention of patients would increase physicians’ consultation fees. Doctors are able to make a profit from individual drug prescriptions, including antibiotics, and this may stimulate over-prescribing of antibiotics.	5 (11.9%)
	lack of pathogen detection or low pathogen detection rate	Uncertainty in the etiological diagnosis is reported as one of the main causes of fear when prescribing in primary care settings. The doctor paid little attention to microbiological examination.	8 (19.0%)
**Caregivers**	lack of skills and knowledge	Parents have considerable misunderstandings that may contribute to inappropriate antibiotic use. Most of parents believe that taking antibiotics in advance could protect children from common diseases.	28 (66.6%)
	put pressure on physician to get antibiotics	Parents’ high expectations of quick relief of symptoms and recovery of their children would impose further pressure on doctors to prescribe antibiotic in order to make treatments more immediately effective.	14 (33.3%)
	self-medicating with antibiotics at home for children	Most of the parents would use lower dose of antibiotics than required by the instruction with consideration of safety, and some parents would choose a higher dose.	14 (33.3%)
**Retail pharmacies**	sale antibiotics without prescription	Although antibiotics sales in retail pharmacies are not within the jurisdiction of government regulation, retail pharmacy is still the main channel for parents to purchase antibiotics.	11 (26.2%)
**Hospitals**	ward capacity	Newborn units with more than 100 beds have the highest rate of antibiotic use, compared to units with 50 or fewer beds, and those with 51–100 beds.	1 (2.4%)

Distribution of disease in children has been regarded as a risk factor influencing antibiotic utilization by 7.1% (3/42) of studies. A study indicated that the reasons that lead physicians prescribe antibiotics were mainly clinical determinants such as severity of symptoms, immediate clinical issue [129]. Another survey on pediatric outpatient prescription found that respiratory infection was one of the diseases with the highest frequency, the most dosage of antibiotic utilization [86]. In addition, children lacking of skills and knowledge about antibiotics also influences antibiotic utilization. Although children knew that antibiotics were not antiviral drugs, they were less able to identify specific antibiotics [121].

A total of 45.2% (19/42) of studies suggested that physicians lacking of skills and knowledge was an important factor influencing antibiotic utilization of children. It was indicated that nearly 30% of pediatricians considered antibiotics to be anti-inflammatories [45], and some village doctors confused to select appropriate antibiotics for children [120]. Some studies explored that pressure from patient had an effect on antibiotic prescriptions. About 70% of the village doctors complied with the primary caregivers’ request even when they felt the antibiotics were unnecessary [120]. Physician-patient relationship was mentioned by 4.8% (2/42) of studies. Physicians wariness of medical disputes by dissatisfied patients might induce them to order unnecessary investigations and overprescribe antibiotics [77]. Economic incentives and profits from prescription also lead physicians to prescribe antibiotics. A study reported that inter-hospital competition was a driver of inappropriate prescribing, if patients did not have antibiotics they want, they will choose other hospital to purchase, leading to suffer financially [128]. A total of 19.0% (8/42) of studies reported that lacking of pathogen detection or low pathogen detection rate was also a risk factor. Through meta-analysis, we found that the overall pathogen detection rate among inpatients was 44.7% (95% *CI*: 29.7-59.7%, *I*^*2*^=99.7%, *P*<0.0001), but there was no study on outpatients. (Fig. S1 in Additional file 1).

A total of 66.6% (28/42) of studies reported that lacking of skills and knowledge from caregivers influences antibiotic utilization for children. Almost 66.3% of respondents mistakenly believed that antibiotics and anti-inflammatory drugs are the same drugs, 68.8% of respondents believed that antibiotics can cure infections caused by virus, 51.5% of respondents believed that antibiotics can be used to treat common cold, 69.9% of respondents believed that antibiotics can be used to treat pharyngitis or nonsuppurative tonsillitis, 37.9% of respondents didn’t know antibiotics should only be obtained with a doctor’s prescription, and 46.6% of respondents didn’t know inappropriate use of antibiotics can reduce the effectiveness of antibiotics (Table S9 in Additional file 1). A total of 33.3% (14/42) of studies reported put pressure on physician to get antibiotics was one of the risk factors influencing antibiotic utilization for children. A study reported that about half of the caregivers had requested antibiotics directly from physicians [117]. A total of 33.3% (14/42) of studies reported self-medicating with antibiotics at home for children was a risk factor of antibiotic utilization. A study reported that about 69.2% of caregivers would self-medication for children before visiting a doctor, in addition, to improve the effectiveness of treatment, they would increase the dosage arbitrarily [118].

For retail pharmacies, selling antibiotics without prescription has been regarded as a risk factor of antibiotic utilization for children. It was reported that individuals in most rural areas continue to have easy access to antibiotics [125]. The rate of antibiotic use was also associated with bed capacity, newborn units with more than 100 beds had the highest rate of antibiotic use, compared to units with 50 or fewer beds, and those with 51–100 beds [124].

## Discussion

The prevalence of antibiotic utilization in Chinese children was high. The overall prevalence of antibiotic utilization among outpatients and inpatients was 63.8% and 81.3%, respectively, and caregivers’ self-medicating with antibiotics for children at home was 37.8%.

Two literature reviews reported a prevalence of 89% or 90.6% for antibiotic utilization in Chinese children, which was higher than that in our study [108, 132]. Compared to before 2010, the prevalence of antibiotics in children has decreased, which was 93.0 % [108, 132]. However, our results were still much higher than the standard values of 20% for outpatient and 60% for inpatient with antibiotic prescriptions issued in an antimicrobial stewardship policy by Chinese government [133]. In addition, the prevalence of antibiotic utilization for children in China was higher than that in the USA (17%-29% ) [134]. According to the ARPEC (Antibiotic Resistance and Prescribing in European Children) report, the prevalence of antibiotic utilization from 226 hospitals in 41 countries was 36.7 % [12]. The overall prevalence of caregiver’s self-medicating of antibiotics for children at home was 37.8%, which was similar to the Chang et.al’ s finding (37.67% ) [122], but lower than that in India (69.4% ) [135].

Children are more susceptible to infection due to their unique peculiarities of body size, surface area, drug metabolism, and excretion, which may be associated with high antibiotic prevalence. However, acute upper respiratory infections (AURIs) are the most common condition associated with the excessive use of antibiotics [136]. Most AURIs are caused by viral infection and usually resolved after three to seven days [137]. However, the use rate of antiviral drugs in the treatment of AURIs in children is low, and most of physicians choose newer, broad-spectrum antibiotics [45]. Therefore, identifying the characteristics of childhood diseases and encouraging the prescriptions of older, narrow-spectrum antibiotics rather than newer, broad-spectrum antibiotics plays an important role in the process of rational use of antibiotics. For school student, there is no systematic teaching on antibiotics, therefore, it is difficult for them to identify antibiotics. In addition, students in different regions have varieties of understandings of antibiotics. The awareness of antibiotics utilization in central urban areas is higher than that of urban and rural areas and counties in suburbs, in addition, the awareness of antibiotics utilization in high school students is higher than that in junior high school and vocational high school [121]. Therefore, more efforts should be made to improve the cognition of student in different regions and different types of school.

The high prevalence of antibiotic utilization in children might have a strong association with physicians, such as lacking of skills and knowledge, pressure from patient, physician-patient relationship, economic incentive and profit from prescribing medicine, lacking of pathogen detection or low pathogen detection rate.

First, physicians in high-level hospitals are more likely to receive training on rational use of antibiotics than those in primary hospitals [138]. Physicians who has not attend related training are more likely to prescribe antibiotics [139]. Sometimes routine training for village doctors is held regularly, however, the content of training is repeated without new course, so the training seems not effective [140]. Therefore, continuous education can narrow the knowledge gap among physicians in different levels of hospital, meanwhile, the quality of education also should be emphasized.

Secondly, many caregivers faithfully embrace the effectiveness of antibiotics and injections over other regimens, and they will actively ask these therapies when visiting doctors. When physicians feel pressure from patients, about 70% of them would prescribe antibiotic [139]. On the other hand, the physician-patient relationship in China is highly strained [141]. Because there is no formal appointment system, patients generally prefer morning consultations, and there is long wait time and short consultations, which cause dissatisfaction among parents [142]. Therefore, some physicians protect themselves by regarding antibiotics as a weapon, especially expensive and injectable ones when they perceive that parents are dissatisfied [77]. They believe that prescribing antibiotics not only satisfy the patients’ claims, but further ease the strained doctor-patient relationship [143].

Thirdly, economic incentive and profit from prescribing medicine also lead physicians to prescribe antibiotics. Antibiotics, which account for approximately 20% of all drug sales in general hospitals [47], are the most frequently used medicine in Chinese medical facilities. In some regions, doctors’ income covered by government funds and profits from drug sales is virtually nail, these changes are not associated with improved antibiotic use. This may partly due to the low salary of medical staff, which motivates the personnel to seek additional income by providing other services and selling pharmaceutical products [144]. In addition, patient retention also could increase the income of physicians. In village clinic, if physicians adopt wait-and-watch policy or prescribe only antiviral drugs for fevers or the common cold, the caregivers would be dissatisfied with the village doctor, and would visit other village doctors. Furthermore, the primary caregivers would not return to the clinic the next time when their child had disease [120]. This may be a reason for the high prevalence of antibiotic utilization in children.

Fourthly, we found that pathogen detection rate among inpatients was 44.7% in our study, which was similar to the result in another study [108]. Antibiotic use without a clear indication is common [126], it is difficult for physicians to distinguish viral infection and bacterial infection, because the diagnostic tests, such as a routine blood test or C-reactive test, are not available in rural settings in China, so they make decisions based on clinical experience without clear indications. However, differentiating definitively between bacterial and viral causes of respiratory infections based on signs and symptoms alone is seldomly possible, and this imprecision and concern about missed bacterial diagnosis likely drives over-prescription of antibiotics [145]. Therefore, additional tools are necessary to physicians.

Parents’ perceptions and practices of how to use medicines have important effect on the management of childhood illness [146]. Through meta-analysis, we found that parents who had considerable misunderstandings may contribute to inappropriate antibiotic use. We found that 66% of caregivers believed that antibiotics could cure infection caused by viruses, a higher percentage than that was found in a pan-European study (54% ) [147]. Furthermore, half of the parents believed that antibiotics could cure common cold. Some parents often overestimate the benefits of antibiotics [148], and consider them as panacea [149]. For caregivers with correct recognition of antibiotics, the self-directed medication rate is lower than the doctor-dependent medication rate [115]. Thus, parents should receive more health education about antibiotics to improve the ability of antibiotic cognition. In addition, one study reported that about 60% of parents asked for doctors to prescribe antibiotics [150], and those who took antibiotic previously were more likely to put pressure on physicians to get them once again [151]. Other inappropriate behaviors include portraying severity of illness, or providing positive experience with use of antibiotic voluntary. However, sometimes, some physicians might misunderstand the willingness of the parents who just would like to ask for an advice or explanation from physicians rather than an antibiotic prescription [152]. Therefore, strategies for effective communication with patients and the prudent prescription of antibiotics are important. In addition, through meta-analysis, we found the overall prevalence of caregiver’s self-medicating of antibiotics for children at home was 37.8%, the result was lower than Yu et.al ’s finding [117]. The reasons for the prevalence of self-medicating children with antibiotics are multifactorial. Storing antibiotics at home increases the probability of medicating children with antibiotics, almost 50% of the caregivers keep antibiotics at home for children [153]. Parents who keep antibiotics at home prefer to self-medicate their children rather than directly seeking advice from a medical professional [122], and people tend to use the same drugs when they confronted similar symptoms based on their experience [154]. In addition, most of home-stored antibiotics are reported to be left over from previous prescriptions [113].

Having purchased antibiotics from retail pharmacies without a physician’s prescription is a critical factor contributing to self-medicating children with antibiotics. As early as 2004, the Chinese government introduced antimicrobial resistance targeted policies, banning the over-the-counter sale of antibiotics [155]. In addition, there is a separate counter displaying antibiotics and labeled “prescription-only medicine” posted to indicating customer antibiotics can only be sold with a prescription. However, in fact, more than 80% pharmacies dispensed antibiotics without a valid prescription. On the one hand, pharmacies dispensed the antibiotics without any prescription; on the other hand, dispensed them with a prescription provided by the pharmacy itself [156]. In addition, in 2012, to supervise the dispensing of medicines and ensure the safety of rational drug use, Chinese government launched the 12th five-year plan on drug safety, which called for all community and hospital pharmacies should have licensed pharmacists on duty during business hours by 201 5[157]. However, when dispensing the antibiotics, most pharmacy staff neither ask information of client nor provide relevant information about antibiotics. Through meta-analysis, we found that about 37% of caregivers do not know that antibiotics should only be obtained with a doctor’s prescription. Although the purchase of antibiotics without a prescription is forbidden by State Food and Drug Administration regulations, customers nevertheless have easy access to antibiotics in most areas. Therefore, stringent implementation of regulations concerning non-prescribed antibiotics in retail pharmacies is essential to restrict access to antibiotics.

Our study has several limitations. First, the heterogeneity across studies was statistically significant and the available data were insufficient to explain all the observed heterogeneity across studies. Second, age groups, hospital wards and characteristics of hospitalized children may also factors influence prevalence of antibiotic prescription. However, it was impossible to conduct meta-analyses due to a smaller number of studies assessing these factors. Third, there are few studies reported the antibiotic utilization in rural areas, however, as there is a rural population of nearly 800 million in China, antibiotic misuse in rural areas may be more serious, and we will investigate antibiotic use among children in rural areas. In addition, only published literatures were included, and potential publication bias cannot be neglected.

## Conclusion

The prevalence of antibiotic utilization is much higher than the standard. The overall prevalence of antibiotic utilization among outpatients, inpatients, and caregiver’s self-medicating at home were 63.8%, 81.3%, and 37.8% respectively. The high prevalence of antibiotics is associated with multiple factors, including at children level (e.g. distribution of disease, lacking of skills and knowledge), and physician level (e.g. lacking of skills and knowledge, pressure from patient, physician-patient relationship, economic incentive and profit from prescribing medicine, lacking of pathogen detection or low pathogen detection rate), and caregiver level (e.g. lacking of skills and knowledge, put pressure on physician to get antibiotics, behavior of self-medicating with antibiotics at home for children) and retail pharmacies level (e.g. sale antibiotics without prescription) and hospital level (e.g. ward capacity). Efforts to improve the prevalence of antibiotic utilization require multisector cooperation, and long-term efforts should target at both children and caregivers, and also prescribers, like health education or training on the proper use of antibiotics and powerful supervision. Further studies should focus on antibiotic utilization of children, especially in rural area.

## Supplementary Information

**Additional file 1:.**

## Data Availability

The datasets analyzed during the current study are not public, but are available from the corresponding author on reasonable request.

## Abbreviations

BRICS: Brazil, Russia, India, China, and South Africa
CARSS: China Antimicrobial Resistance Surveillance System
NASP: National Antibiotic Stewardship Program
PRISMA: Preferred Reporting Items for Systematic Reviews and Meta-Analyses
CNKI: China National Knowledge Infrastructure
CI: Confidence interval
AHRQ: Agency for Healthcare Research and Quality
ARPEC: Antibiotic Resistance and Prescribing in European Children
AURIs: Acute upper respiratory infections
