# Supplementary material for: Prevalence and risk factors for antibiotic utilization in Chinese children
Source: BMC Pediatr. 2021 Jun 1;21:255. doi: 10.1186/s12887-021-02706-z (PMC8168021; doi:10.1186/s12887-021-02706-z)
Supplement: Supplementary file 1 — Additional file 1:. [file 12887_2021_2706_MOESM1_ESM.docx]

**Appendix A**

Table S1**.** The Preferred Reporting Items for Systematic Reviews and Meta-Analyses (PRISMA) 2009 Checklist. [1]

| **Section/topic** | **#** | **Checklist item** | **Reported on page #** | |
| --- | --- | --- | --- | --- |
| **TITLE** | | |  | |
| Title | 1 | Identify the report as a systematic review, meta-analysis, or both. | 1 | |
| **ABSTRACT** | | |  | |
| Structured summary | 2 | Provide a structured summary including, as applicable: background; objectives; data sources; study eligibility criteria, participants, and interventions; study appraisal and synthesis methods; results; limitations; conclusions and implications of key findings; systematic review registration number. | 1 | |
| **INTRODUCTION** | | |  | |
| Rationale | 3 | Describe the rationale for the review in the context of what is already known. | 2 | |
| Objectives | 4 | Provide an explicit statement of questions being addressed with reference to participants, interventions, comparisons, outcomes, and study design (PICOS). | 2 | |
| **METHODS** | | |  | |
| Protocol and registration | 5 | Indicate if a review protocol exists, if and where it can be accessed (e.g., Web address), and, if available, provide registration information including registration number. | 2 | |
| Eligibility criteria | 6 | Specify study characteristics (e.g., PICOS, length of follow-up) and report characteristics (e.g., years considered, language, publication status) used as criteria for eligibility, giving rationale. | 3 | |
| Information sources | 7 | Describe all information sources (e.g., databases with dates of coverage, contact with study authors to identify additional studies) in the search and date last searched. | 2-3 | |
| Search | 8 | Present full electronic search strategy for at least one database, including any limits used, such that it could be repeated. | 2-3 | |
| Study selection | 9 | State the process for selecting studies (i.e., screening, eligibility, included in systematic review, and, if applicable, included in the meta-analysis). | 3 | |
| Data collection process | 10 | Describe method of data extraction from reports (e.g., piloted forms, independently, in duplicate) and any processes for obtaining and confirming data from investigators. | 3 | |
| Data items | 11 | List and define all variables for which data were sought (e.g., PICOS, funding sources) and any assumptions and simplifications made. | 3 | |
| Risk of bias in individual studies | 12 | Describe methods used for assessing risk of bias of individual studies (including specification of whether this was done at the study or outcome level), and how this information is to be used in any data synthesis. | 3 | |
| Summary measures | 13 | State the principal summary measures (e.g., risk ratio, difference in means). | 3 | |
| Synthesis of results | 14 | Describe the methods of handling data and combining results of studies, if done, including measures of consistency (e.g., I^2^) for each meta-analysis. | 3 | |
| Risk of bias across studies | 15 | Specify any assessment of risk of bias that may affect the cumulative evidence (e.g., publication bias, selective reporting within studies). | 8 | |
| Additional analyses | 16 | Describe methods of additional analyses (e.g., sensitivity or subgroup analyses, meta-regression), if done, indicating which were pre-specified. | 3 | |
| **RESULTS** | | |  |  |
| Study selection | 17 | Give numbers of studies screened, assessed for eligibility, and included in the review, with reasons for exclusions at each stage, ideally with a flow diagram. | 3 | |
| Study characteristics | 18 | For each study, present characteristics for which data were extracted (e.g., study size, PICOS, follow-up period) and provide the citations. | Appendix S2 & S3 & S4 & S5 | |
| Risk of bias within studies | 19 | Present data on risk of bias of each study and, if available, any outcome level assessment (see item 12). | Appendix S6 & S7 | |
| Results of individual studies | 20 | For all outcomes considered (benefits or harms), present, for each study: (a) simple summary data for each intervention group (b) effect estimates and confidence intervals, ideally with a forest plot. | 4 | |
| Synthesis of results | 21 | Present results of each meta-analysis done, including confidence intervals and measures of consistency. | 4 | |
| Risk of bias across studies | 22 | Present results of any assessment of risk of bias across studies (see Item 15). | 8 | |
| Additional analysis | 23 | Give results of additional analyses, if done (e.g., sensitivity or subgroup analyses, meta-regression [see Item 16]). | 3-5 | |
| **DISCUSSION** | | |  |  |
| Summary of evidence | 24 | Summarize the main findings including the strength of evidence for each main outcome; consider their relevance to key groups (e.g., healthcare providers, users, and policy makers). | 5-7 | |
| Limitations | 25 | Discuss limitations at study and outcome level (e.g., risk of bias), and at review-level (e.g., incomplete retrieval of identified research, reporting bias). | 7 | |
| Conclusions | 26 | Provide a general interpretation of the results in the context of other evidence, and implications for future research. | 8 | |
| **FUNDING** | | |  |  |
| Funding | 27 | Describe sources of funding for the systematic review and other support (e.g., supply of data); role of funders for the systematic review. | 8 | |

**Appendix B**

**Table S2.** Search strategies to identify studies reporting the prevalence and risk factor for antibiotic utilization in children in China.

1. **English database**
2. ***Pubmed***

| Search | Query | Items found |
| --- | --- | --- |
| #7 | Search: ((((anti-bacterial agents [MeSH Terms]) AND (antibiot*[Title/Abstract] OR antimicrobial*[Title/Abstract] OR anti-biotic[Title/Abstract] OR anti-microbial[Title/Abstract] OR anti-bacterial[Title/Abstract] OR agents, anti-bacterial[Title/Abstract] OR antibacterial agents[Title/Abstract])) AND (((epidemiology [MeSH Terms]) OR (prevalence rate [Title/Abstract] OR epidemic [Title/Abstract] OR epidemiological investigation[Title/Abstract] OR distribution[Title/Abstract] OR rate*[Title/Abstract] OR ratio[Title/Abstract] OR proport*[Title/Abstract] OR percent*[Title/Abstract])) OR ((risk factor[MeSH Terms]) OR (cause OR drive OR driving OR predict* OR determinant* OR mechanism OR risk factor)))) AND ((infant OR child OR adolescent[MeSH Terms]) OR (preschool[Title/Abstract] OR child*[Title/Abstract] OR adolesen*[Title/Abstract] OR teen*[Title/Abstract] OR youth*[Title/Abstract] OR neonat*[Title/Abstract] OR pre-school[Title/Abstract] OR new-born [Title/Abstract]))) AND (China[Title/Abstract] OR Chinese[Title/Abstract]) | 479 |
| #6 | Search: China [Title/Abstract] OR Chinese [Title/Abstract] | 348,637 |
| #5 | Search: (infant OR child OR adolescent [MeSH Terms]) OR (preschool [Title/Abstract] OR child*[Title/Abstract] OR adolesen*[Title/Abstract] OR teen*[Title/Abstract] OR youth*[Title/Abstract] OR neonat*[Title/Abstract] OR pre-school [Title/Abstract] OR new-born [Title/Abstract]) | 4,316,709 |
| #4 | Search: ((epidemiology [MeSH Terms]) OR (prevalence rate [Title/Abstract] OR epidemic [Title/Abstract] OR epidemiological investigation [Title/Abstract] OR distribution [Title/Abstract] OR rate*[Title/Abstract] OR ratio [Title/Abstract] OR proport*[Title/Abstract] OR percent*[Title/Abstract])) OR ((risk factor [MeSH Terms]) OR (cause OR drive OR driving OR predict* OR determinant* OR mechanism OR risk factor)) | 15,657,921 |
| #3 | Search: (risk factor [MeSH Terms]) OR (cause OR drive OR driving OR predict* OR determinant* OR mechanism OR risk factor) | 13,136,935 |
| #2 | Search: (epidemiology [MeSH Terms]) OR (prevalence rate [Title/Abstract] OR epidemic [Title/Abstract] OR epidemiological investigation [Title/Abstract] OR distribution [Title/Abstract] OR rate*[Title/Abstract] OR ratio [Title/Abstract] OR proport*[Title/Abstract] OR percent*[Title/Abstract]) | 5,238,710 |
| #1 | Search: (anti-bacterial agents [MeSH Terms]) AND (antibiot*[Title/Abstract] OR antimicrobial*[Title/Abstract] OR anti-biotic [Title/Abstract] OR anti-microbial [Title/Abstract] OR anti-bacterial [Title/Abstract] OR agents, anti-bacterial [Title/Abstract] OR antibacterial agents [Title/Abstract]) | 197,830 |

1. ***Web of Science***

| Search | Query | Items found |
| --- | --- | --- |
| #7 | #6 AND #5 AND #4 AND #1 | 2,450 |
| #6 | TS= (China OR Chinese) | 1,761,831 |
| #5 | TS= (infant OR preschool OR child* OR adolesen* OR teen* OR youth* OR neonat* OR pre-school OR new-born) | 4,650,903 |
| #4 | #3 OR #2 | 29,814,295 |
| #3 | TS= (risk factor OR cause OR drive OR driving OR predict* OR determinant* OR mechanism) | 17,088,808 |
| #2 | TS= (epidemiology OR prevalence rate OR epidemic OR epidemiological investigation OR distribution OR rate* OR ratio OR proport* OR percent*) | 18,213,422 |
| #1 | TS= (antibiot* OR antibacterial OR antimicrobial* OR anti-biotic OR anti-microbial OR anti-bacterial) | 1,288,160 |

1. ***Embase***

| Search | Query | Items found |
| --- | --- | --- |
| #7 | #1 AND #4 AND #5 AND #6 | 547 |
| #6 | china:ab,ti OR chinese:ab,ti | 431,292 |
| #5 | infant:ab,ti OR preschool:ab,ti OR child*:ab,ti OR adolesen*:ab,ti OR teen*:ab,ti OR youth*:ab,ti OR neonat*:ab,ti OR 'pre school':ab,ti OR 'new born':ab,ti | 2,307,826 |
| #4 | #2 OR #3 | 10,592,493 |
| #3 | 'risk factor':ab,ti OR cause:ab,ti OR drive:ab,ti OR driving:ab,ti OR predict*:ab,ti OR determinant*:ab,ti OR mechanism:ab,ti | 5,198,010 |
| #2 | epidemiology:ab,ti OR 'prevalence rate':ab,ti OR epidemic:ab,ti OR 'epidemiological investigation':ab,ti OR distribution:ab,ti OR rate*:ab,ti OR ratio:ab,ti OR proport*:ab,ti OR percent*:ab,ti | 6,979,968 |
| #1 | antibiot*:ab,ti OR antibacterial:ab,ti OR antimicrobial*:ab,ti OR 'anti biotic':ab,ti OR 'anti microbial':ab,ti OR 'anti bacterial':ab,ti | 672,477 |

1. **Chinese database**

| Database | | Query | Items |
| --- | --- | --- | --- |
| Chongqing VIP | M=(抗生素 OR 抗菌素 OR 抗菌药 OR 抗微生物) AND M=(影响因素 OR 危险因素 OR 决定因素 OR 高危因素 OR 原因 OR 率 OR 流行病 OR 分布) AND M=(婴儿 OR 新生儿 OR 幼儿 OR 小儿 OR 患儿 OR 儿童 OR 青少年 OR 未成年) | 385 |  |
| CNKI | (KY % '抗生素'+'抗菌素'+'抗菌药'+'抗微生物') AND (SU % '影响因素'+ '危险因素'+'决定因素'+'高危因素'+'原因'+'率'+'流行率'+'分布') AND (SU % '婴儿'+ '新生儿'+ '幼儿'+ '小儿'+ '患儿'+ '儿童'+ '青少年'+ '未成年') | 2,450 |  |
| Wan Fang | 题名或关键词:(抗生素 OR 抗菌素 OR 抗菌药 OR 抗微生物)*主题:(影响因素 OR 危险因素 OR 决定因素 OR 高危因素 OR 原因 OR 率 OR 流行病 OR 分布)*主题:(婴儿 OR 新生儿 OR 幼儿 OR 小儿 OR 患儿 OR 儿童 OR 青少年 OR 未成年) | 3,662 |  |

CNKI: China National Knowledge Infrastructure

**Appendix C**

**Table S3(1).** Detailed characteristics of the included articles (outpatient, n=35).

| **Study**  **(Publication Year)** | **Study Period** | **Province** | **Geographical Region** | **Setting** | **Hospital Levels** | **Sample Size (N)** | **Age** | **Number of Children with Antibiotics(n)** | **Number of Children with Single Antibiotic** | **Number of Children with Combined Antibiotic** |
| --- | --- | --- | --- | --- | --- | --- | --- | --- | --- | --- |
| Liang X, et al. (2010)[2] | 2009-2010 | Guangdong | eastern | urban | 3 | 5585 | 0-14 | 5362 | 4466 | 896 |
| Hu X (2010)[3] | 2010 | Chongqing | western | urban | 2 | 859 | 0-12 | 611 | 298 | 313 |
| Chen C, et al. (2010)[4] | 2010 | Guangdong | eastern | urban | 3 | 1000 | 0-14 | 833 | 649 | 184 |
| Zhang J (2011)[5] | 2010 | Tianjin | eastern | urban | 3 | 6996 | 0-14 | 3541 | 3460 | 81 |
| Wang J, et al. (2011)[6] | 2010 | Guangdong | eastern | urban | 3 | 1000 | 0-14 | 799 | 439 | 360 |
| Li X (2011)[7] | 2009-2010 | Hunan | central | urban | 2 | 756 | 0-14 | 522 | 489 | 33 |
| Liang J, et al. (2012)[8] | 2010 | Guangdong | eastern | urban | 1 | 500 | 0-15 | 354 | 146 | 208 |
| Huang X, et al. (2012)[9] | 2012 | Jiangsu | eastern | urban | 3 | 194 | 0-14 | 62 | NA | NA |
| Li H (2012)[10] | 2010 | Shandong | eastern | urban | 3 | 2111 | 0-14 | 1326 | NA | NA |
| Chen L (2012)[11] | 2012 | Jiangsu | eastern | urban | 3 | 6687 | 0-14 | 3423 | NA | NA |
| Zhou H, et al. (2012)[12] | 2011 | Fujian | eastern | urban | 3 | 3360 | 0-14 | 1441 | 1279 | 162 |
| Li L, et al. (2013)[13] | 2011-2012 | Shanxi(陕西) | western | urban | 3 | 1272 | 0-14 | 1015 | 965 | 50 |
| Xiao B (2013)[14] | 2011 | Sichuan | western | urban | 3 | 3256 | 0-14 | 1553 | 1366 | 187 |
| Chen H, et al. (2013)[15] | 2012 | Guangdong | eastern | urban | 2 | 4172 | 3m-5 | 315 | NA | NA |
| Liu H (2013) [16] | 2012-2014 | Hebei | eastern | urban | 2 | 2178 | 0-8 | 814 | 198 | 616 |
| Miao C, et al. (2014)[17] | 2012 | Guangdong | eastern | urban | 3 | 1000 | 0-14 | 457 | NA | NA |
| Zeng Y (2014)[18] | 2012 | Hunan | central | urban | 3 | 1230 | 0-14 | 788 | NA | NA |
| Gui M, et al. (2014)[19] | 2010 | Henan | central | urban | 2 | 1000 | 0-5 | 1000 | 873 | 127 |
| Gui M, et al. (2014)[19] | 2011 | Henan | central | urban | 2 | 1000 | 0-5 | 960 | 907 | 53 |
| Gui M, et al. (2014)[19] | 2012 | Henan | central | urban | 2 | 1000 | 0-5 | 879 | 879 | 0 |
| Yuan Y, et al. (2015)[20] | 2010-2011 | Beijing | eastern | urban | 3 | 37211 | 0-18 | 21864 | NA | NA |
| Xu P, et al. (2015)[21] | 2014 | Shandong | eastern | urban | 1 | 600 | 0-14 | 586 | 337 | 249 |
| Xu X, et al. (2015)[22] | 2014-2015 | Guangdong | eastern | urban | 1 | 300 | 2m-12 | 120 | 106 | 14 |
| Gao Y, et al. (2016)[23] | 2015 | Xinjiang | western | rural | 1 | 566 | 2-12 | 513 | 513 | 0 |
| Xie Y (2016)[24] | 2015 | Anhui | central | urban | 3 | 258 | 1-13 | 214 | NA | NA |
| Lin Y, et al. (2016)[25] | 2014-2015 | Yunnan | western | urban | 3 | 600 | 0-14 | 430 | 430 | 0 |
| Wang J (2016)[26] | 2011-2014 | Hebei | eastern | urban | 3 | 1300 | 0-11 | 1020 | NA | NA |
| Wang S (2017)[27] | 2016 | Jiangsu | eastern | urban | 3 | 28069 | 0-14 | 15692 | NA | NA |
| Pan Y (2017)[28] | 2016 | Shanghai | eastern | urban | 3 | 1000 | 0-14 | 532 | 532 | 0 |
| Nie C, et al. (2017)[29] | 2017 | Yunnan | western | urban | 2 | 1087 | 0-14 | 511 | 494 | 17 |
| Zhang Z, et al. (2017)[30] | 2014 | Guangxi | eastern | rural | 2 | 8166 | 2-14 | 2736 | 1438 | 1298 |
| Zhang Z, et al. (2017)[30] | 2014 | Guangxi | eastern | rural | 1 | 877 | 2-14 | 593 | 506 | 87 |
| Wei X, et al. (2017)[31] | 2015-2016 | Guangxi | eastern | rural | 1 | 2349 | 2-14 | 1936 | 1661 | 275 |
| Wei X, et al. (2017)[31] | 2015-2016 | Guangxi | eastern | rural | 1 | 2548 | 2-14 | 1922 | 1667 | 255 |
| Wei X, et al. (2017)[32] | 2014 | Guangxi | eastern | rural | 2 | 4452 | 2-14 | 399 | 369 | 30 |
| Wei X, et al. (2017)[32] | 2011 | Guangxi | eastern | rural | 2 | 1809 | 2-14 | 1590 | 717 | 873 |
| Wei X, et al. (2017)[32] | 2014 | Guangxi | eastern | rural | 2 | 2333 | 2-14 | 2018 | 759 | 1259 |
| Wei X, et al. (2017)[32] | 2011 | Guangxi | eastern | rural | 2 | 3766 | 2-14 | 1148 | 1098 | 50 |
| Wang Z, et al. (2018)[33] | 2016 | Jiangsu | eastern | rural | 1 | 466 | 0-16 | 304 | NA | NA |
| Wang C (2019)[34] | 2015-2016 | Shanxi(陕西) | western | urban | 2 | 900 | 0-14 | 738 | 617 | 121 |
| Wei X, et al. (2019)[35] | 2015 | Guangxi | eastern | rural | 1 | 1400 | 2-14 | 1063 | NA | NA |
| Wei X, et al. (2019)[35] | 2015 | Guangxi | eastern | rural | 1 | 1400 | 2-14 | 1171 | NA | NA |
| Peng L, et al. (2019)[36] | 2017-2018 | Shandong | eastern | urban | 3 | 1541 | 0-14 | 1199 | 713 | 486 |

NA: not available

**Table S3(2).** Detailed characteristics of the included articles (outpatient, n=35).

| **Study** **(Publication Year)** | **Study Period** | **Drug.1 ATC Code (%)** | **Drug.2 ATC Code (%)** | **Drug.3 ATC Code (%)** |
| --- | --- | --- | --- | --- |
| Liang X, et al. (2010)[2] | 2009-2010 | cephalosporins-J01D  56.1 | penicillinss-J01C 36.9 | NA |
| Hu X (2010)[3] | 2010 | penicillinss-J01C 56.5 | cephalosporins-J01D  53.2 | NA |
| Chen C, et al. (2010)[4] | 2010 | cephalosporins-J01D  84.0 | macrolides-J01FA 23.3 | lincosamides 7.8 |
| Zhang J (2011)[5] | 2010 | Oral:  azithromycin-J01FA10 44.7 Intravenous: ceftezole-J01DB12 43.8 | Oral: cefaclor-J01DC04 14.7 Intravenous: azlocillin-J01CA09 14.8 | Oral: cefadroxil-J01DB05 13.1 Intravenous: cefoxitin-J01DC01 11.3 |
| Wang J, et al. (2011)[6] | 2010 | Oral:  clindamycin-J01FF01 38.0 Intravenous: cefamandole-J01DC03 28.0 | Oral: azithromycin-J01FA10 18.0 Intravenous: cefotaxime-J01DD01 19.0 | Oral: cefprozil-J01DC10 16.0 Intravenous: ceftizoxime-J01DD07 15.0 |
| Li X (2011)[7] | 2009-2010 | azithromycin-J01FA10 26.9 | ceftriaxone-J01DD54 21.0 | cefuroxime-J01DC02 12.1 |
| Liang J, et al. (2012)[8] | 2010 | cefradine-J01DB09 36.0 | clindamycin-J01FF01 21.0 | azithromycin-J01FA10 15.0 |
| Huang X, et al. (2012)[9] | 2012 | NA | NA | NA |
| Li H (2012)[10] | 2010 | cephalosporins-J01D | penicillinss-J01C | macrolides-J01FA |
| Chen L (2012)[11] | 2012 | aztreonam-J01DF01 | cefoxitin-J01DC01 | NA |
| Zhou H, et al. (2012)[12] | 2011 | cephalosporins-J01D  82.6 | macrolides-J01FA 14.3 | penicillinss-J01C 2.2 |
| Li L, et al. (2013)[13] | 2011-2012 | NA | NA | NA |
| Xiao B (2013)[14] | 2011 | cephalosporins-J01D  48.0 | macrolides-J01FA 25.6 | penicillinss-J01C 21.8 |
| Chen H, et al. (2013)[15] | 2012 | cefprozil-J01DC10 | azithromycin-J01FA10 | clindamycin-J01FF01 |
| Liu H (2013)[16] | 2012-2014 | cefazolin-J01DB04 21.3 | cefmenoxime-J01DD05 13.5 | azithromycin-J01FA10 12.2 |
| Miao C, et al. (2014)[17] | 2012 | azithromycin-J01FA10 33.9 | cefixime-J01DD08 23.7 | cefaclor-J01DC04 12.3 |
| Zeng Y (2014)[18] | 2012 | NA | cefuroxime-J01DC02 | ceftriaxone-J01DD54 |
| Gui M, et al. (2014)[19] | 2010 | azlocillin-J01CA09 | amoxicillin-J01CA04 | mezlocillin-J01CA10 |
| Gui M, et al. (2014) [19] | 2011 | azlocillin-J01CA09 | amoxicillin-J01CA04 | mezlocillin-J01CA10 |
| Gui M, et al. (2014) [19] | 2012 | cefuroxime-J01DC02 | cefaclor-J01DC04 | amoxicillin-J01CA04 |
| Yuan Y, et al. (2015)[20] | 2010-2011 | Oral:  cephalosporins-J01D  Intravenous: penicillinss-J01C | Oral: macrolides-J01FA Intravenous: macrolides-J01FA | Oral: penicillinss-J01C Intravenous: NA |
| Xu P, et al. (2015)[21] | 2014 | penicillinss-J01C 29.9 | cephalosporins-J01D  15.0 | macrolides-J01FA 6.14 |
| Xu X, et al. (2015)[22] | 2014-2015 | cephalosporins-J01D  11.3 | penicillinss-J01C 9.0 | NA |
| Gao Y, et al. (2016)[23] | 2015 | cefuroxime-J01DC02 60.0 | cefotaxime-J01DD01 16.0 | clindamycin-J01FF01 11.0 |
| Xie Y (2016)[24] | 2015 | clindamycin-J01FF01 | cefpodoxime-J01DD13 | azithromycin-J01FA10 |
| Lin Y, et al. (2016)[25] | 2014-2015 | NA | NA | NA |
| Wang J (2016)[26] | 2011-2014 | azithromycin-J01FA10 | ceftazidime-J01DD02 | cefuroxime-J01DC02 |
| Wang S (2017)[27] | 2016 | Oral:  third-generation cephalosporins-J01DD Intravenous: penicillinss-J01C | NA | NA |
| Pan Y (2017)[28] | 2016 | cefaclor-J01DC04 36.8 | cefprozil-J01DC10 14.3 | cefdinir-J01DD15 11.7 |
| Nie C, et al. (2017)[29] | 2017 | Oral:  amoxicillin-J01CA04 20.65 Intravenous: amoxicillin-J01CA04 42.9 | Oral: cefaclor-J01DC04 5.87 Intravenous: ceftazidime-J01DD02 12.35 | Oral: azithromycin-J01FA10 2.43 Intravenous: cefuroxime-J01DC02 4.86 |
| Zhang Z, et al. (2017)[30] | 2014 | third-generation cephalosporins-J01DD 47.5 | second-generation cephalosporins-J01DC 45.9 | first-generation cephalosporins-J01DB 18.0 |
| Zhang Z, et al. (2017) [30] | 2014 | first-generation cephalosporins-J01DB 16.2 | third-generation cephalosporins-J01DD 12.6 | second-generation cephalosporins-J01DC 9.6 |
| Zhang Z, et al. (2017) [30] | 2014 | aminoglycoside antibiotics-J01G 33.33 | penicillins-J01C 33.33 | first-generation cephalosporins-J01DB 29.2 |
| Wei X, et al. (2017) [31] | 2015-2016 | NA | NA | NA |
| Wei X, et al. (2017) [31] | 2015-2016 | NA | NA | NA |
| Wei X, et al. (2017) [32] | 2014 | NA | NA | NA |
| Wei X, et al. (2017) [32] | 2011 | NA | NA | NA |
| Wei X, et al. (2017) [32] | 2014 | NA | NA | NA |
| Wei X, et al. (2017) [32] | 2011 | NA | NA | NA |
| Wang Z, et al. (2018) [33] | 2016 | Oral:  cefixime-J01DD08 8.6 Intravenous: azithromycin-J01FA10 16.7 | Oral: azithromycin-J01FA10 5.5 Intravenous: cefotaxime-J01DD01 13.3 | Oral: amoxicillin-J01CA04 3.0 Intravenous: ceftizoxime-J01DD07 12.6 |
| Wang C (2019) [34] | 2015-2016 | azithromycin-J01FA10 | ceftazidime-J01DD02 | cefaclor-J01DC04 |
| Wei X, et al. (2019) [35] | 2015 | NA | NA | NA |
| Wei X, et al. (2019) [35] | 2015 | NA | NA | NA |
| Peng L, et al. (2019) [36] | 2017-2018 | ceftriaxone-J01DD54 | cefmetazole-J01DC09 | metronidazole-J01XD01 |

NA: not available

**Table S4(1).** Detailed characteristics of the included articles (inpatient, n=41).

| **Study**  **(Publication Year)** | **Study Period** | **Province** | | **Geographical Region** | | **Setting** | | **Hospital Levels** | | **Sample Size (N)** | | **Age** | **Number of Children with Antibiotics(n)** | | **Number of Children with Single Antibiotic** | | **Number of Children with Antibiotic Combination** | |  |
| --- | --- | --- | --- | --- | --- | --- | --- | --- | --- | --- | --- | --- | --- | --- | --- | --- | --- | --- | --- |
| Wei X, et al. (2017) [32] | 2011 | Guangxi | eastern | | rural | | 2 | | 74 | | 2-14 | | | 47 | | NA | | NA | |
| Wei X, et al. (2017) [32] | 2014 | Guangxi | eastern | | rural | | 2 | | 51 | | 2-14 | | | 38 | | NA | | NA | |
| Wei X, et al. (2017) [32] | 2011 | Guangxi | eastern | | rural | | 2 | | 59 | | 2-14 | | | 49 | | NA | | NA | |
| Wei X, et al. (2017) [32] | 2014 | Guangxi | eastern | | rural | | 2 | | 67 | | 2-14 | | | 42 | | NA | | NA | |
| Cui Y (2011)[37] | 2010-2011 | Sichuan | western | | urban | | 3 | | 1560 | | 3m-15 | | | 1017 | | 538 | | 479 | |
| Qiu Z, et al. (2011)[38] | 2009-2011 | Guangdong | eastern | | urban | | 3 | | 200 | | 1m-13 | | | 195 | | 65 | | 130 | |
| Chen J, et al. (2011)[39] | 2010 | Guangdong | eastern | | urban | | 3 | | 341 | | 1m-14 | | | 328 | | NA | | NA | |
| Lin Y, et al. (2011) [40] | 2008-2010 | Hainan | eastern | | urban | | 3 | | 132 | | 9m-14 | | | 52 | | 48 | | 4 | |
| Liang Z, et al. (2011)[41] | 2009-2010 | Guangdong | eastern | | urban | | 3 | | 180 | | 1d-12 | | | 122 | | 40 | | 82 | |
| Zhou L (2012)[42] | 2011 | Guangxi | eastern | | urban | | 2 | | 426 | | 0-14 | | | 293 | | 293 | | 0 | |
| Bian H, et al. (2012)[43] | 2010-2011 | Jiangsu | eastern | | urban | | 3 | | 360 | | 0-14 | | | 320 | | 179 | | 141 | |
| Fang J (2012)[44] | 2010 | Sichuan | western | | urban | | 2 | | 196 | | 0-14 | | | 193 | | 141 | | 52 | |
| Wu W (2012)[45] | 2011 | Sichuan | western | | urban | | 3 | | 2466 | | 1m-13 | | | 2424 | | 898 | | 1526 | |
| He M (2012)[46] | 2009-2011 | Hunan | central | | urban | | 2 | | 360 | | 2-12 | | | 326 | | 98 | | 228 | |
| Guan L (2012)[47] | 2010 | Anhui | central | | urban | | 3 | | 628 | | 1m-14 | | | 467 | | 428 | | 39 | |
| Jiang L, et al. (2012)[48] | 2011 | Xinjiang | western | | urban | | 3 | | 1148 | | 0-16 | | | 1087 | | 709 | | 378 | |
| Zhu J, et al. (2012)[49] | 2007-2010 | Yunnan | western | | urban | | 3 | | 7400 | | 1-28d | | | 2675 | | NA | | NA | |
| Wang Q, et al. (2012)[50] | 2011 | Shandong | eastern | | urban | | 3 | | 1289 | | 0-17 | | | 1264 | | 945 | | 319 | |
| Che Y, et al. (2013)[51] | 2010-2011 | Shanxi(陕西) | western | | urban | | 2 | | 447 | | 1-30 days | | | 395 | | 32 | | 363 | |
| Lu J, et al. (2013)[52] | 2011-2013 | Guangxi | eastern | | urban | | 3 | | 2258 | | 0-14 | | | 1867 | | 410 | | 1457 | |
| Xu J (2013)[53] | 2012 | Zhejiang | eastern | | urban | | 3 | | 1188 | | 2m-13 | | | 1099 | | 1040 | | 59 | |
| Huang X, et al. (2013)[54] | 2011 | Sichuan | western | | urban | | 3 | | 215 | | 0-14 | | | 199 | | 136 | | 63 | |
| Zhan J, et al. (2013)[55] | 2012-2013 | Guangdong | eastern | | urban | | 2 | | 360 | | 3-5 | | | 325 | | 69 | | 256 | |
| Li C, et al. (2013)[56] | 2010 | Nationwide | nationwide | | urban | | 3 | | 3607 | | 1-28d | | | 2724 | | NA | | NA | |
| Li C, et al. (2013)[56] | 2010 | Nationwide | nationwide | | urban | | 3 | | 9874 | | 1-18 | | | 8113 | | NA | | NA | |
| Yao Q, et al. (2014)[57] | 2010-2012 | Anhui | central | | urban | | 3 | | 800 | | 0-14 | | | 700 | | 349 | | 351 | |
| Wu M, et al. (2014)[58] | 2012 | Guangdong | eastern | | urban | | 2 | | 240 | | 0-14 | | | 227 | | 85 | | 142 | |
| Zhu G, et al. (2014)[59] | 2012 | Jiangsu | eastern | | urban | | 3 | | 1269 | | 34d-4 | | | 1132 | | 708 | | 424 | |
| Liu Y (2014)[60] | 2014 | Guizhou | western | | urban | | 2 | | 240 | | 0-14 | | | 205 | | NA | | NA | |
| Huang W (2014)[61] | 2013 | Guangxi | eastern | | urban | | 3 | | 395 | | 0-13 | | | 347 | | 285 | | 62 | |
| Huang G, et al. (2014)[62] | 2011 | Guangxi | eastern | | urban | | 3 | | 1139 | | 1-28d | | | 876 | | 774 | | 102 | |
| Cheng X, et al. (2014)[63] | 2013 | Guangdong | eastern | | urban | | 3 | | 297 | | 1m-14 | | | 258 | | 146 | | 112 | |
| Cheng H, et al. (2014)[64] | 2013 | Hubei | central | | urban | | 3 | | 1352 | | 0-14 | | | 1306 | | 814 | | 492 | |
| Chen Y, et al. (2015)[65] | 2014 | Jiangsu | eastern | | urban | | 3 | | 2286 | | 1-28d | | | 2216 | | NA | | NA | |
| Li L, et al. (2017)[66] | 2013-2014 | Hebei | eastern | | urban | | 2 | | 68 | | 0-2 | | | 65 | | NA | | NA | |
| Su X, et al. (2017)[67] | 2008-2011 | Neimenggu | central | | urban | | 3 | | 800 | | 0-14 | | | 795 | | 699 | | 96 | |
| Su X, et al. (2017)[67] | 2012-2015 | Neimenggu | central | | urban | | 3 | | 800 | | 0-14 | | | 615 | | 563 | | 52 | |
| Zhu J, et al. (2017)[68] | 2015 | Guangdong | eastern | | urban | | 3 | | 3349 | | 1-28d | | | 2271 | | NA | | NA | |
| Li R, et al. (2018)[69] | 2016 | Shanxi(山西) | central | | urban | | 3 | | 1000 | | 3-14 | | | 454 | | NA | | NA | |
| Chen Z, et al. (2018)[70] | 2016 | Tianjin | eastern | | urban | | 3 | | 918 | | 0-10 | | | 454 | | 314 | | 140 | |
| Zhang J, et al. (2018)[71] | 2016-2017 | Nationwide | nationwide | | urban | | 3 | | 1439 | | 1-18 | | | 975 | | 722 | | 253 | |
| Zhang J, et al. (2018)[71] | 2016-2017 | Nationwide | nationwide | | urban | | 3 | | 298 | | 1-28d | | | 173 | | 123 | | 50 | |
| Ju J, et al. (2019)[72] | 2016-2018 | Shandong | eastern | | urban | | 3 | | 236 | | 0-15 | | | 139 | | 58 | | 81 | |
| Wei W, et al. (2019)[73] | 2014-2016 | Nationwide | nationwide | | urban | | 3 | | 1383 | | 0-14 | | | 1232 | | 963 | | 269 | |
| Niu J (2020)[74] | 2018-2019 | Ningxia | western | | urban | | 2 | | 850 | | 1m-12 | | | 790 | | 650 | | 140 | |
| Miao R, et al. (2020)[75] | 2018 | Sichuan | western | | urban | | 3 | | 881 | | 1-14 | | | 406 | | NA | | NA | |
| Miao R, et al. (2020)[75] | 2018 | Sichuan | western | | urban | | 3 | | 323 | | 1-14 | | | 285 | | NA | | NA | |
| Miao R, et al. (2020)[75] | 2018 | Sichuan | western | | urban | | 2 | | 382 | | 1-14 | | | 334 | | NA | | NA | |
| Miao R, et al. (2020)[75] | 2018 | Sichuan | western | | urban | | 2 | | 127 | | 1-14 | | | 108 | | NA | | NA | |
| Miao R, et al. (2020)[75] | 2018 | Sichuan | western | | rural | | 1 | | 45 | | 1-14 | | | 43 | | NA | | NA | |
| Zhang M, et al. (2020)[76] | 2015 | Hebei | eastern | | urban | | 3 | | 766 | | 0-18 | | | 701 | | 477 | | 224 | |
| Zhang M, et al. (2020)[76] | 2016 | Hebei | eastern | | urban | | 3 | | 696 | | 0-14 | | | 645 | | 287 | | 358 | |
| Zhang M, et al. (2020)[76] | 2017 | Hebei | eastern | | urban | | 3 | | 862 | | 0-14 | | | 788 | | 368 | | 420 | |
| Zhang M, et al. (2020)[76] | 2018 | Hebei | eastern | | urban | | 3 | | 657 | | 0-14 | | | 611 | | 260 | | 351 | |

NA: not available

**Table S4(2).** Detailed characteristics of the included articles (inpatient, n=41).

| **Study** **(Publication Year)** | **Study Period** | **Drug.1 ATC Code (%)** | **Drug.2 ATC Code (%)** | **Drug.3 ATC Code (%)** |
| --- | --- | --- | --- | --- |
| Wei X, et al. (2017) [32] | 2014 | NA | NA | NA |
| Wei X, et al. (2017) [32] | 2011 | NA | NA | NA |
| Wei X, et al. (2017) [32] | 2014 | NA | NA | NA |
| Wei X, et al. (2017) [32] | 2011 | NA | NA | NA |
| Cui Y (2011)[37] | 2010-2011 | penicillins-J01C | cephalosporins-J01D | macrolides-J01FA |
| Qiu Z, et al. (2011)[38] | 2009-2011 | cephalosporins-J01D | penicillins-J01C | macrolides-J02FA |
| Chen J, et al. (2011)[39] | 2010 | ceftriaxone-J01DD54 22.6 | mezlocillin-J01CA10 19.7 | ceftazidime-J01DD02 13.8 |
| Lin Y, et al. (2011)[40] | 2008-2010 | azithromycin-J01FA10  32.7 | cefoperazone-J01DD12 21.2 | cefuroxime-J01DC02 19.2 |
| Liang Z, et al. (2011)[41] | 2009-2010 | ceftriaxone-J01DD54 | cefoperazone-J01DD12 | ceftazidime-J01DD02 |
| Zhou L (2012)[42] | 2011 | cefuroxime-J01DC02 32.3 | ceftazidime-J01DD02 25.7 | ceftriaxone-J01DD54 19.3 |
| Bian H, et al. (2012)[43] | 2010-2011 | penicillins-J01C 49.1 | third-generation cephalosporins-J01DD 43.5 | macrolides-J01FA 18.1 |
| Fang J (2012)[44] | 2010 | second-generation cephalosporins-J01DC | third-generation cephalosporins-J01DD | penicillins-J01C |
| Wu W (2012)[45] | 2011 | cephalosporins-J01D  53.6 | penicillins-J01C  40.4 | NA |
| He M (2012)[46] | 2009-2011 | cefoperazone-J01DD12 | cefuroxime-J01DC02 | amoxicillin-J01CA04 |
| Guan L (2012)[47] | 2010 | NA | NA | NA |
| Jiang L, et al. (2012)[48] | 2011 | NA | NA | NA |
| Zhu J, et al. (2012)[49] | 2007-2010 | NA | NA | NA |
| Wang Q, et al. (2012)[50] | 2011 | cefuroxime-J01DC02 19.5 | amoxicillin-J01CA04  18.8 | azithromycin-J01FA10 14.9 |
| Che Y, et al. (2013)[51] | 2010-2011 | penicillins-J01C | cephalosporins-J01D | macrolides-J01FA |
| Lu J, et al. (2013)[52] | 2011-2013 | piperacillin-J01CA12 24.3 | cefoperazone-J01DD12 16.0 | ceftezole-J01DB12 12.0 |
| Xu J (2013)[53] | 2012 | ceftriaxone-J01DD54 | azithromycin-J01FA10 | cefprozil-J01DC10 |
| Huang X, et al. (2013)[54] | 2011 | [cefathiamidine](javascript:;) | [amoxicillin and beta-lactamase inhibitor](https://db.yaozh.com/atc?atc_num=J01CR02) | cefuroxime-J01DC02 |
| Zhan J, et al. (2013)[55] | 2012-2013 | cephalosporins-J01D  52.3 | penicillins-J01C 25.6 | macrolides-J01FA 25.8 |
| Li C, et al. (2013)[56] | 2010 | NA | NA | NA |
| Li C, et al. (2013) [56] | 2010 | NA | NA | NA |
| Yao Q, et al. (2014)[57] | 2010-2012 | ceftizoxime-J01DD07 21.0 | [amoxicillin and beta-lactamase inhibitor 11.9](https://db.yaozh.com/atc?atc_num=J01CR02) | azithromycin-J01FA10 8.36 |
| Wu M, et al. (2014)[58] | 2012 | cephalosporins-J01D | penicillins-J01C | macrolides-J01FA |
| Zhu G, et al. (2014)[59] | 2012 | ceftizoxime-J01DD07 64.3 | benzylpenicillin-J01CE01 46.8 | azithromycin-J01FA10 36.7 |
| Liu Y (2014)[60] | 2014 | benzylpenicillin-J01CE01 31.1 | mezlocillin-J01CA10 24.8 | cefuroxime-J01DC02 17.0 |
| Huang W (2014)[61] | 2013 | piperacillin-J01CA12 36.6 | ceftazidime-J01DD02 13.0 | azithromycin-J01FA10 9.8 |
| Huang G, et al. (2014)[62] | 2011 | ceftazidime-J01DD02 40.0 | mezlocillin-J01CA10 36.4 | piperacillin-J01CA12 24.7 |
| Cheng X, et al. (2014)[63] | 2013 | cefoxitin-J01DC01 35.7 | ceftazidime-J01DD02 31.8 | [erythromycin-J01FA01 29.8](https://db.yaozh.com/atc?atc_num=J01FA01) |
| Cheng H, et al. (2014)[64] | 2013 | ceftriaxone-J01DD54 | cefaclor-J01DC04 | azithromycin-J01FA10 |
| Chen Y, et al. (2015)[65] | 2014 | NA | NA | NA |
| Li L, et al. (2017)[66] | 2013-2014 | NA | NA | NA |
| Su X, et al. (2017)[67] | 2008-2011 | third-generation cephalosporins-J01DD 40.4 | second-generation cephalosporins-J01DC 36.9 | NA |
| Su X, et al. (2017) [67] | 2012-2015 | second-generation cephalosporins-J01DC 31.8 | third-generation cephalosporins-J01DD 16.7 | NA |
| Zhu J, et al. (2017)[68] | 2015 | benzylpenicillin-J01CE01 | ceftazidime-J01DD02 | [erythromycin-J01FA01](https://db.yaozh.com/atc?atc_num=J01FA01) |
| Li R, et al. (2018)[69] | 2016 | penicillins-J01C 38.1 | cephalosporins-J01D 25.6 | gentamicin-J01GB03 11.5 |
| Chen Z, et al. (2018)[70] | 2016 | latamoxef-J01DD06 29.3 | azithromycin-J01FA10 19.0 | ceftriaxone-J01DD54  12.9 |
| Zhang J, et al. (2018)[71] | 2016-2017 | third-generation cephalosporins-J01DD 35.5 | macrolides-J01FA 23.2 | beta-lactam antibacterial, penicillin -J01C 15.9 |
| Zhang J, et al. (2018) [71] | 2016-2017 | third-generation cephalosporins-J01DD 41.7 | beta-lactam antibacterial, penicillin -J01C 23.8 | Carbapenems-J01DH 11.2 |
| Ju J, et al. (2019)[72] | 2016-2018 |  |  |  |
| Wei W, et al. (2019)[73] | 2014-2016 | cephalosporins-J01D  38.3 | macrolides-J01FA 55.8 | NA |
| Niu J (2020)[74] | 2018-2019 | NA | NA | NA |
| Miao R, et al. (2020)[75] | 2018 | cefoperazone and enzyme inhibitors | meloxicillin and enzyme inhibitor | azithromycin-J01FA10 |
| Miao R, et al. (2020) [75] | 2018 | ceftazidime-J01DD02 | cefathiamidine | cefuroxime-J01DC02 |
| Miao R, et al. (2020) [75] | 2018 | cefixime-J01DD08 | cefoperazone and enzyme inhibitors | azithromycin-J01FA10 |
| Miao R, et al. (2020) [75] | 2018 | cefoxitin-J01DC01 | cefsulodin-J01DD03 | NA |
| Miao R, et al. (2020) [75] | 2018 | cefoperazone and enzyme inhibitors | cefoxitin-J01DC01 | azithromycin-J01FA10 |
| Zhang M, et al. (2020)[76] | 2015 | azithromycin-J01FA10 72.3 | ceftezole-J01DB12 48.1 | cefuroxime-J01DC02 23.1 |
| Zhang M, et al. (2020)[76] | 2016 | azithromycin-J01FA10 60.8 | ceftezole-J01DB12 48.5 | erythromycin-J01FA01 32.9 |
| Zhang M, et al. (2020)[76] | 2017 | azithromycin-J01FA10 57.3 | cefoxitin-J01DC01 46.2 | ceftezole-J01DB12 37.0 |
| Zhang M, et al. (2020)[76] | 2018 | cefoxitin-J01DC01 59.0 | azithromycin-J01FA10 53.8 | erythromycin-J01FA01 36.8 |

NA: not available

**Table S5.** Detailed characteristics of the included articles (caregiver’s self-medicating of antibiotics for children at home, n=4).

| **Study (Publication Year)** | **Study Period** | **Province** | **Setting** | **Sample Size (N)** | **Number of Children with Antibiotics(n)** |
| --- | --- | --- | --- | --- | --- |
| Cui F, et al. (2015)[77] | 2011 | Beijing | Hospital | 1412 | 218 |
| Zhao M, et al. (2016)[78] | NA | Shanxi(陕西) | School | 1052 | 767 |
| Yin Y, et al. (2018)[79] | 2016 | Shandong | Hospital and School | 362 | 154 |
| Peng D, et al. (2018)[80] | NA | Zhejiang | School | 556 | 113 |

NA: not available

**Table S6.** Detailed characteristics of the included articles (risk factors, n=42).

| **Study**  **(Publication Year)** | **Province** | **No. of participants** | **Data Sources** | **Authors' conclusions and summary of key findings** |
| --- | --- | --- | --- | --- |
| Liang X, et al. (2010)[2] | Guangdong | 5585 pieces of transfusion prescriptions | outpatient department | The infusion antibiotics usage rate account for 96% of survey prescriptions. Repeated use of antibiotics is one of the risk factors of unreasonable use of drugs. The physiological characteristics of children determine the drug use. |
| Hu X (2010)[3] | Chongqing | 859 pieces of prescriptions | outpatient department | The utilization of antibiotics in pediatric outpatient department is reasonable. However, there are some problems, including physicians prescribe antibiotics based on practical experience, no clear indication of drug utilization and unreasonable antibiotic combination, etc. |
| Huang X, et al. (2012)[9] | Jiangsu | 194 pieces of prescriptions | outpatient department | The utilization of antibiotics in pediatric outpatient department is reasonable. Distribution of disease, the physiological characteristics of children are associated with high utilization of antibiotics. |
| Zhou H, et al. (2012)[12] | Fujian | 3360 prescriptions | outpatient department | Physicians prescribed antibiotics based on practical experience, and the traditional misunderstanding that family members regard antibiotics as "panacea" has contributed to the high utilization rate of pediatric antibiotics. The special physiological characteristics of children make it difficult to use pediatric drugs rationally in clinical practice. |
| Li L, et al. (2013)[13] | Shanxi(陕西) | 1272 copies of upper respiratory tract infection prescriptions | outpatient department | Antibiotics continue to be widely prescribed to treat upper respiratory tract infection. The cognition of rational drug us in clinicians is low, and they prescribe antibiotics based on practical experience. |
| Gui M, et al. (2014)[19] | Henan | 3000 cases of prescription with upper respiratory tract infection | outpatient department | For the upper respiratory tract infection caused mostly by virus, no pharyngeal swab bacterial culture and drug sensitivity test were performed, and there was no indication of using antibiotics, physicians prescribed antibiotics based on practical experience. Parents lack of knowledge about antibiotics, and clinicians did not attach importance to rational antibiotic use. |
| Yuan Y, et al. (2015)[20] | Beijing | 37211 prescriptions with upper respiratory infection | outpatient department | Antibiotics prescription rate are high for children with upper respiratory infections, particularly the prescription of broad-spectrum antibiotics and intravenous antibiotics. |
| Lin Y, et al. (2016)[25] | Yunnan | 600 prescriptions for upper respiratory tract infection | outpatient department | Viral infection and bacterial infection are similar in early clinical manifestations, and some clinicians tend to prophylactic medication for the purpose of "double insurance". Doctors and parents lack of knowledge about the adverse reactions of intravenous infusion. |
| Wang S (2017)[27] | Jiangsu | 28069 cases of respiratory tract infection | outpatient department | Most parents believe that antibiotics have a therapeutic effect on upper respiratory tract infection; at present, the doctor-patient relationship is highly strained, and outpatient doctors are afraid of taking risks and applying antibiotics. |
| Wang C (2019)[34] | Shanxi(陕西) | 900 cases with acute upper respiratory tract infection | outpatient department | Immunofluorescence detection is difficult to operate for some physicians. Some parents are eager to see a doctor and blindly ask doctors use drugs in a rapid and large dose in order to increase the curative effect, which easily leads to the abuse of antibiotics. |
| Qiu Z, et al. (2011)[38] | Guangdong | 200 cases with acute upper respiratory tract infection. | inpatient department | The phenomenon of irrational use of antibiotics for acute upper respiratory tract infection exists. There is lack of pathogen detection for children with acute upper respiratory tract infection; the caregivers’ expectations to antibiotics is associated with whether an antibiotic is prescribed. |
| Fang J (2012)[44] | Sichuan | 196 cases with acute upper respiratory tract infection | inpatient department | The use of antibiotics in pediatric patients with acute upper respiratory infection is a severe problem. Doctors’ understanding to etiology of acute upper respiratory tract infection in children is obscure; doctors believe that antibiotics are necessary to treat upper respiratory infections; parents lack of knowledge about antibiotics. |
| Wu W (2012)[45] | Sichuan | 2466 cases of infection | inpatient department | High prevalence of antibiotic utilization among hospitalized children was found in the study. The doctor-patient relationship is extremely tense, and pediatricians have been used antibiotics as a weapon to protect themselves. |
| Che Y, et al. (2013)[51] | Shanxi(陕西) | 447 medical records of neonates | primary hospital | The utilization of antibiotics of children is unreasonable. Doctors lack of awareness to use antibiotics rationally. Parents lack of knowledge about antibiotics, and ask doctors for antibiotics proactively. Retail pharmacies sale antibiotics without prescription. |
| Lu J, et al. (2013)[52] | Guangxi | 2258 cases with acute upper respiratory tract infection | inpatient department | There is a high rate of antibiotic use and low pathogens rate of children with acute respiratory tract infection. Pediatricians lack of knowledge about antibiotics. |
| Xu J (2013)[53] | Zhejiang | 1188 pieces of prescriptions | inpatient department | The prevalence of antibiotics use in inpatient department is high. Clinicians payed little attention to the use of antimicrobials reasonably. |
| Zhu G, et al. (2014)[59] | Jiangsu | 1269 medical records | inpatient department | There is a high rate of antibiotic use of children in inpatient department. Children have lower immune resistance than adults. Misunderstanding to antibiotics of pediatricians and children's parents exists. |
| Liu Y (2014)[60] | Guizhou | 240 medical records | inpatient department | The doctor did not adjust the antibiotics usage according to the conditions of the microbiological examination and the results of the examination. |
| Su X, et al. (2017)[67] | Neimenggu | 800 cases of pediatric bronchial pneumonia | inpatient department | Caregivers regarded antibiotics as panacea and retail pharmacies sale antibiotics without prescription. Community physicians who did not have the conditions or experience to test for pathogens prescribed antibiotics. |
| Cui F, et al. (2015)[77] | Beijing | 1500 parents of children | three hospitals | Currently antibiotics abuse still exists to some extent. The antibiotic application of the parents of children indicated cognitive deficiencies. |
| Zhao M, et al. (2016)[78] | Shanxi(陕西) | 1052 children' caregivers | a kindergarten and a primary school | There are still many problems in rational use of antibiotics in children. The age, occupation and monthly income of the interviewees may be the factors influencing parents' self-medication with antibiotics in the children． |
| Yin Y, et al. (2018)[79] | Shandong | 362 parents of children | 5 hospitals and 2 kindergartens | There are abuse of antibiotics and overuse of intravenous antibiotic administration among preschool children. In addition, the parents of the children have limited knowledge about antibiotic use and its adverse effect and they do not consider indications of antibiotics administrated to their children． |
| Peng D, et al. (2018)[80] | Zhejiang | 2806 parents of children | a kindergarten and a primary school | Poor knowledge and massive antibiotic misuse for children among parents are of a great concern. Parents with city residence, higher education level and medical background were more likely to store antibiotics at home; parents with city residence and those store antibiotics at home were more likely to self-medicate their children with antibiotics. |
| Yao Z, et al. (2013)[81] | Guangzhou | 1295 children' caregivers | kindergarten | The prevalence of self-medication with antibiotics for kindergarten children is high. The educational level of parents and antibiotics kept at home were the main determinants of self-medication with antibiotics in the children． |
| Miao R (2013)[82] | Sichuan | 539 pupils' parents | primary school | There were serious problems in the cognitive behaviors of pupils' parents about antibiotic use. Home-reserving antibiotics was the risk factor for parents using antibiotics independently. Parental age and medical professional of the family were protective factors. |
| Zhang G, et al. (2014)[83] | Beijing | 2055 children' caregivers | pediatrics medicine clinic | The level of parent’s antibiotic knowledge had been slightly improved in that 10 years. Parents’ compliance to doctor about antibiotic’s application had decreased between that 10 years. There was no significant change on the parents’ knowledge about antibiotic abuse will reduce therapy effects and will lead to bacteria resistance in that decade. |
| Yu M, et al. (2014)[84] | Jiangxi | 854 children' caregivers | clinics for the vaccination | Low levels of knowledge on the use of antibiotics and a high prevalence of self-medicating children with antibiotics were observed among parents in rural China. Living in rural villages, raising more than one child, increasing age of child, purchasing antibiotics without a prescription, storing antibiotics at home and good adherence to physicians’ advice were independently associated with self-medicating behavior. |
| Ding L, et al. (2016)[85] | Shandong | 727 children' caregivers | 12 villages in Yanggu | The rural parents’ cognition on rational use of antibiotics needs to be improved by strengthening popularity. Its main influencing factors were educational background and age of caregivers, and the relationship between caregivers and their children． |
| Cen Q, et al. (2016)[86] | Hubei | 80 novice mothers | community service center | Novice mothers are generally short of knowledge about the use of antibiotics, especially of knowledge about use of drugs for treating viral infections. There are errors in administration of the antibiotics and the cognitive level of novice mothers to the dangers of abusing antibiotics is limited. |
| Li R, et al. (2016)[87] | Nationwide | 53665 guardians of children | community health centers | There has been a high rate of antibiotic misuse without a prescription in children with diarrhea in China. Urban area, female children, higher education of guardians, being raised by parents, guardians having basic health knowledge were protective factors and children's age, 4-6 years was risk factor of antibiotic misuse among children. |
| Zhang Z, et al. (2016)[88] | Qinghai | 35 village doctors, 13 primary caregivers, 17 directors of township hospitals, county-level health bureaus, county-level Centers for Disease Control and Prevention, or county-level Chinese Food and Drug Administration offices. | NA | Most of the village doctors had inadequate knowledge and misconceptions about antibiotic use, which was an important factor in the unnecessary prescribing. Village doctors and directors reported that the doctors’ fear of complications, the primary caregivers’ pressure for antibiotic treatment, and the financial considerations of patient retention were the main factors influencing the decision to prescribe antibiotics. |
| Wang J, et al. (2017)[89] | Hunan | 310 parents of young children | community health service centers | The knowledge about rational use of antibiotics needs to be promoted among the parents of young children. The parents’ behavior of buying antibiotics from drug stores for the medication of their children are influenced by the age of their children, education level of themselves, and whether their children having been prescribed with antibiotics by doctors. |
| Zhang Y, et al. (2017)[90] | Shanxi(陕西) | 250 parents of young children | rural residents | The awareness rate of antibiotics in the guardians of children aged 0-6 years in the rural area is low, and guardians with low educational level as well as grandparents as the caregivers are the key groups. |
| Cheng Y, et al. (2017)[91] | Beijing | 4220 students | nine middle schools | The cognition of drug safety in middle school students is good, but problems still exist in medication adherence, the management of expired drugs and the antibiotics cognition. |
| Chang J, et al. (2018)[92] | Shanghai Hunan Shanxi(陕西) | 4200 caregivers of children | preschool | There was a high proportion of primary caregivers self-medicate antibiotics for children in urban China, despite their insufficient knowledge about antibiotic use. |
| Fan W, et al. (2019)[93] | Tianjin | 400 parents of children | 3 hospitals and 3 communities | There was a relatively high degree of antibiotic cognition among parents of preschool children in Tianjin, but there were misunderstandings in the cognition and use principles of antibiotics. Antibiotic use in children was 77.5%. |
| Ge Y, et al. (2019)[94] | Nationwide | 55 newborn units | 25 provinces | Antibiotic use rates were significantly different by newborn ward bed capacity. Newborn units with more than 100 beds had the highest rate of antibiotic use. |
| Cheng J, et al. (2019)[95] | Anhui | 960 households with children | respondents’ homes | Children with upper respiratory tract infection had a high consultation rate and a high prescription rate of antibiotics. For the children, some caregivers adopted, without rationale, ‘self -medication’ with antibiotics. Children with fever were more likely to be taken to a doctor and children with particular symptoms were more likely to receive a prescription for antibiotics. |
| Wang J, et al. (2019)[96] | Shanghai | 1368 young parents | three community healthcare centers | Antibiotics use is widespread over all three study sites. Lack of knowledge and poor perception of antibiotics usage are found among young parents. |
| Ye D, et al. (2020)[97] | Shanxi(陕西) | 472 pediatricians | online | The pediatricians' age, education level, and monthly income and whether had ever received training had significant associations with their knowledge level. Uncertain diagnosis, parent requirements and insufficient time were barriers to appropriate antibiotic prescription. |
| Wei X, et al. (2020)[98] | Guangxi | hospital directors, doctors, and caregivers of children | Rong County and Liujiang County | Intervention-arm doctors described that training sessions improved their knowledge, skills and confidence in appropriate prescribing. Caregiver participants reported that intervention educational materials were helpful but they identified information from doctors was more useful. Providers and caregivers also described contextual health system factors, including hospital competition, short consultation times, and antibiotic availability without prescription, which shaped care preferences. |
| Zhang J, et al. (2020)[99] | Asia-Pacific countries | 731 physicians | online | Chinese physicians were significantly more concerned about safety profile and antibiotic resistance when deciding for antibiotic prescription in acute respiratory symptoms with fever. |

**Appendix D**

**Table S7.** Quality scores for assessing the risk of bias of in the included articles for observation study.

| **Score**  **Study (Year)** | **Q1** | **Q2** | **Q3** | **Q4** | **Q5** | **Q6** | **Q7** | **Q8** | **Q9** | **Q10** | **Q11** | **Total Score** |
| --- | --- | --- | --- | --- | --- | --- | --- | --- | --- | --- | --- | --- |
| Liang X, et al. (2010)[2] | 1 | 0 | 1 | 1 | 1 | 0 | 0 | 0 | 0 | 1 | 1 | 6 |
| Hu X (2010)[3] | 1 | 0 | 1 | 1 | 1 | 0 | 0 | 0 | 0 | 1 | 1 | 6 |
| Chen C, et al. (2010) [4] | 1 | 1 | 1 | 1 | 1 | 0 | 0 | 0 | 0 | 1 | 1 | 7 |
| Zhang J (2011)[5] | 1 | 0 | 1 | 1 | 1 | 0 | 0 | 0 | 0 | 1 | 1 | 6 |
| Wang J, et al. (2011)[6] | 1 | 0 | 1 | 1 | 1 | 0 | 0 | 0 | 0 | 1 | 1 | 6 |
| Li X (2011)[7] | 1 | 1 | 1 | 1 | 1 | 0 | 1 | 0 | 0 | 1 | 1 | 8 |
| Liang J, et al. (2012)[8] | 1 | 0 | 1 | 1 | 1 | 0 | 0 | 0 | 0 | 1 | 1 | 6 |
| Huang X, et al. (2012)[9] | 1 | 0 | 1 | 1 | 1 | 0 | 0 | 0 | 0 | 1 | 1 | 6 |
| Li H (2012)[10] | 1 | 0 | 1 | 1 | 1 | 0 | 0 | 0 | 0 | 1 | 1 | 6 |
| Chen L (2012)[11] | 1 | 1 | 1 | 1 | 1 | 0 | 0 | 0 | 0 | 1 | 1 | 7 |
| Zhou H, et al. (2012)[12] | 1 | 1 | 1 | 1 | 1 | 0 | 1 | 1 | 0 | 1 | 1 | 9 |
| Li L, et al. (2013)[13] | 1 | 1 | 1 | 1 | 1 | 0 | 1 | 1 | 0 | 1 | 1 | 9 |
| Xiao B (2013)[14] | 1 | 1 | 1 | 1 | 1 | 0 | 0 | 0 | 0 | 1 | 1 | 7 |
| Chen H, et al. (2013)[15] | 1 | 1 | 1 | 1 | 1 | 0 | 0 | 1 | 0 | 1 | 1 | 8 |
| Liu H (2013)[16] | 1 | 1 | 1 | 1 | 1 | 0 | 1 | 1 | 0 | 1 | 1 | 9 |
| Miao C, et al. (2014)[17] | 1 | 0 | 1 | 1 | 1 | 0 | 0 | 0 | 0 | 1 | 1 | 6 |
| Zeng Y (2014)[18] | 1 | 0 | 1 | 1 | 1 | 0 | 0 | 0 | 0 | 1 | 1 | 6 |
| Gui M, et al. (2014)[19] | 1 | 1 | 1 | 1 | 1 | 0 | 0 | 0 | 0 | 1 | 1 | 7 |
| Yuan Y, et al. (2015)[20] | 1 | 1 | 1 | 1 | 1 | 0 | 1 | 1 | 0 | 1 | 1 | 9 |
| Xu P, et al. (2015)[21] | 1 | 0 | 1 | 1 | 1 | 0 | 0 | 0 | 0 | 1 | 1 | 6 |
| Xu X, et al. (2015)[22] | 1 | 1 | 1 | 1 | 1 | 0 | 1 | 1 | 0 | 1 | 1 | 9 |
| Gao Y, et al. (2016)[23] | 1 | 0 | 1 | 1 | 1 | 0 | 0 | 0 | 0 | 1 | 1 | 6 |
| Lin Y, et al. (2016)[25] | 1 | 0 | 1 | 1 | 1 | 0 | 0 | 0 | 0 | 1 | 1 | 6 |
| Wang J (2016)[26] | 1 | 0 | 1 | 1 | 1 | 0 | 0 | 0 | 0 | 1 | 1 | 6 |
| Wang S (2017)[27] | 1 | 1 | 1 | 1 | 1 | 0 | 1 | 0 | 0 | 1 | 1 | 8 |
| Pan Y (2017)[28] | 1 | 1 | 1 | 1 | 1 | 0 | 0 | 0 | 0 | 1 | 1 | 7 |
| Nie C, et al. (2017)[29] | 1 | 1 | 1 | 1 | 1 | 0 | 0 | 0 | 0 | 1 | 1 | 7 |
| Zhang Z, et al. (2017)[30] | 1 | 1 | 1 | 1 | 1 | 0 | 1 | 1 | 0 | 1 | 1 | 9 |
| Wei X, et al. (2017)[32] | 1 | 1 | 1 | 1 | 1 | 0 | 1 | 1 | 0 | 1 | 1 | 9 |
| Wang Z, et al. (2018)[33] | 1 | 1 | 1 | 1 | 1 | 0 | 0 | 0 | 0 | 1 | 1 | 7 |
| Wang C (2019)[34] | 1 | 0 | 1 | 1 | 1 | 0 | 0 | 0 | 0 | 1 | 1 | 6 |
| Peng L, et al. (2019)[36] | 1 | 1 | 1 | 1 | 1 | 0 | 1 | 1 | 0 | 1 | 1 | 9 |
| Cui Y (2011)[37] | 1 | 0 | 1 | 1 | 1 | 0 | 0 | 0 | 0 | 1 | 1 | 6 |
| Qiu Z, et al. (2011)[38] | 1 | 0 | 1 | 1 | 1 | 0 | 0 | 0 | 0 | 1 | 1 | 6 |
| Chen J, et al. (2011)[39] | 1 | 1 | 1 | 1 | 1 | 0 | 0 | 0 | 0 | 1 | 1 | 7 |
| Lin Y, et al. (2011)[40] | 1 | 0 | 1 | 1 | 1 | 0 | 0 | 0 | 0 | 1 | 1 | 6 |
| Liang Z, et al. (2011)[41] | 1 | 1 | 1 | 1 | 1 | 0 | 0 | 0 | 0 | 1 | 1 | 7 |
| Zhou L (2012)[42] | 1 | 1 | 1 | 1 | 1 | 0 | 0 | 0 | 0 | 1 | 1 | 7 |
| Bian H, et al. (2012)[43] | 1 | 1 | 1 | 1 | 1 | 0 | 0 | 0 | 0 | 1 | 1 | 7 |
| Fang J (2012)[44] | 1 | 1 | 1 | 1 | 1 | 0 | 0 | 0 | 0 | 1 | 1 | 7 |
| Wu W (2012)[45] | 1 | 0 | 1 | 1 | 1 | 0 | 0 | 0 | 0 | 1 | 1 | 6 |
| He M (2012)[46] | 1 | 1 | 1 | 1 | 1 | 0 | 0 | 0 | 0 | 1 | 1 | 7 |
| Guan L (2012)[47] | 1 | 0 | 1 | 1 | 1 | 0 | 0 | 0 | 0 | 1 | 1 | 6 |
| Jiang L, et al. (2012)[48] | 1 | 1 | 1 | 1 | 1 | 0 | 1 | 1 | 0 | 1 | 1 | 9 |
| Zhu J, et al. (2012)[49] | 1 | 1 | 1 | 1 | 1 | 0 | 1 | 0 | 0 | 1 | 1 | 8 |
| Wang Q, et al. (2012)[50] | 1 | 1 | 1 | 1 | 1 | 0 | 0 | 0 | 0 | 1 | 1 | 7 |
| Che Y, et al. (2013)[51] | 1 | 1 | 1 | 1 | 1 | 0 | 0 | 0 | 0 | 1 | 1 | 7 |
| Lu J, et al. (2013)[52] | 1 | 1 | 1 | 1 | 1 | 0 | 0 | 0 | 0 | 1 | 1 | 7 |
| Xu J (2013)[53] | 1 | 1 | 1 | 1 | 1 | 0 | 0 | 0 | 0 | 1 | 1 | 7 |
| Huang X, et al. (2013)[54] | 1 | 1 | 1 | 1 | 1 | 0 | 0 | 0 | 0 | 1 | 1 | 7 |
| Zhan J, et al. (2013)[55] | 1 | 0 | 1 | 1 | 1 | 0 | 0 | 0 | 0 | 1 | 1 | 6 |
| Li C, et al. (2013)[56] | 1 | 1 | 1 | 1 | 1 | 0 | 1 | 1 | 0 | 1 | 1 | 9 |
| Yao Q, et al. (2014)[57] | 1 | 0 | 1 | 1 | 1 | 0 | 0 | 0 | 0 | 1 | 1 | 6 |
| Wu M, et al. (2014)[58] | 1 | 1 | 1 | 1 | 1 | 0 | 0 | 0 | 0 | 1 | 1 | 7 |
| Zhu G, et al. (2014)[59] | 1 | 0 | 1 | 1 | 1 | 0 | 0 | 0 | 0 | 1 | 1 | 6 |
| Liu Y (2014)[60] | 1 | 0 | 1 | 1 | 1 | 0 | 0 | 0 | 0 | 1 | 1 | 6 |
| Huang W (2014)[61] | 1 | 1 | 1 | 1 | 1 | 0 | 0 | 0 | 0 | 1 | 1 | 7 |
| Huang G, et al. (2014)[62] | 1 | 1 | 1 | 1 | 1 | 0 | 0 | 0 | 0 | 1 | 1 | 7 |
| Cheng X, et al. (2014)[63] | 1 | 0 | 1 | 1 | 1 | 0 | 0 | 0 | 0 | 1 | 1 | 6 |
| Cheng H, et al. (2014)[64] | 1 | 1 | 1 | 1 | 1 | 0 | 0 | 0 | 0 | 1 | 1 | 7 |
| Chen Y, et al. (2015)[65] | 1 | 0 | 1 | 1 | 1 | 0 | 0 | 0 | 0 | 1 | 1 | 6 |
| Su X, et al. (2017)[67] | 1 | 1 | 1 | 1 | 1 | 0 | 1 | 0 | 0 | 1 | 1 | 8 |
| Li R, et al. (2018)[69] | 1 | 0 | 1 | 1 | 1 | 0 | 0 | 0 | 0 | 1 | 1 | 6 |
| Chen Z, et al. (2018)[70] | 1 | 1 | 1 | 1 | 1 | 0 | 0 | 0 | 0 | 1 | 1 | 7 |
| Zhang J, et al. (2018)[71] | 1 | 1 | 1 | 1 | 1 | 0 | 1 | 0 | 0 | 1 | 1 | 8 |
| Wei W, et al. (2019)[73] | 1 | 1 | 1 | 1 | 1 | 0 | 1 | 1 | 0 | 1 | 1 | 9 |
| Niu J (2020)[74] | 1 | 0 | 1 | 1 | 1 | 0 | 0 | 0 | 0 | 1 | 1 | 6 |
| Miao R, et al. (2020)[75] | 1 | 1 | 1 | 1 | 1 | 0 | 0 | 1 | 0 | 1 | 1 | 8 |
| Zhang M, et al. (2020)[76] | 1 | 1 | 1 | 1 | 1 | 1 | 0 | 0 | 0 | 1 | 1 | 8 |
| Cui F, et al. (2015)[77] | 1 | 1 | 1 | 1 | 1 | 1 | 0 | 1 | 0 | 1 | 1 | 9 |
| Zhao M, et al. (2016)[78] | 1 | 0 | 1 | 1 | 1 | 0 | 0 | 0 | 0 | 1 | 1 | 6 |
| Yin Y, et al. (2018)[79] | 1 | 1 | 1 | 1 | 1 | 0 | 0 | 1 | 0 | 1 | 1 | 8 |
| Peng D, et al. (2018)[80] | 1 | 1 | 1 | 1 | 1 | 0 | 0 | 1 | 0 | 1 | 1 | 8 |
| Yao Z, et al. (2013)[81] | 1 | 1 | 1 | 1 | 1 | 1 | 0 | 1 | 0 | 1 | 1 | 9 |
| Miao R (2013)[82] | 1 | 0 | 0 | 1 | 1 | 1 | 0 | 1 | 0 | 1 | 1 | 7 |
| Zhang G, et al. (2014)[83] | 1 | 1 | 1 | 1 | 1 | 1 | 0 | 1 | 0 | 1 | 1 | 9 |
| Yu M, et al. (2014)[84] | 1 | 1 | 1 | 1 | 1 | 1 | 0 | 1 | 0 | 1 | 1 | 9 |
| Ding L, et al. (2016)[85] | 1 | 1 | 1 | 1 | 1 | 1 | 0 | 1 | 0 | 1 | 1 | 9 |
| Cen Q, et al. (2016)[86] | 1 | 0 | 1 | 1 | 1 | 1 | 0 | 0 | 0 | 1 | 1 | 7 |
| Li R, et al. (2016)[87] | 1 | 1 | 1 | 1 | 1 | 0 | 0 | 1 | 0 | 1 | 1 | 8 |
| Zhang Z, et al. (2016)[88] | 1 | 1 | 1 | 1 | 1 | 0 | 0 | 1 | 0 | 1 | 1 | 8 |
| Wang J, et al. (2017)[89] | 1 | 1 | 1 | 1 | 1 | 0 | 0 | 1 | 0 | 1 | 1 | 8 |
| Zhang Y, et al. (2017)[90] | 1 | 0 | 1 | 1 | 1 | 0 | 0 | 0 | 0 | 1 | 1 | 6 |
| Cheng Y, et al. (2017)[91] | 1 | 0 | 1 | 1 | 1 | 0 | 0 | 1 | 0 | 1 | 1 | 7 |
| Chang J, et al. (2018)[92] | 1 | 1 | 1 | 1 | 1 | 1 | 0 | 1 | 0 | 1 | 1 | 9 |
| Fan W, et al. (2019)[93] | 1 | 1 | 1 | 1 | 1 | 0 | 0 | 0 | 0 | 1 | 1 | 7 |
| Ge Y, et al. (2019)[94] | 1 | 0 | 1 | 1 | 1 | 1 | 0 | 1 | 0 | 1 | 1 | 8 |
| Cheng J, et al. (2019)[95] | 1 | 1 | 1 | 1 | 1 | 0 | 0 | 1 | 0 | 1 | 1 | 8 |
| Wang J, et al. (2019)[96] | 1 | 1 | 1 | 1 | 1 | 1 | 0 | 1 | 0 | 1 | 1 | 9 |
| Ye D, et al. (2020)[97] | 1 | 1 | 1 | 1 | 1 | 0 | 0 | 1 | 0 | 1 | 1 | 8 |
| Wei X, et al. (2020)[98] | 1 | 1 | 1 | 1 | 1 | 0 | 0 | 1 | 0 | 1 | 1 | 8 |
| Zhang J, et al. (2019)[99] | 1 | 1 | 1 | 1 | 1 | 0 | 0 | 1 | 0 | 1 | 1 | 8 |

“No” or “Unclear” was scored “0”, and “Yes” was scored “1”. ≤3 scores: low quality; 4-7 scores: moderate quality; ≥8 scores: high quality. Q1: Define the source of information (survey, record review); Q2: List inclusion and exclusion criteria for exposed and unexposed subjects (cases and controls) or refer to previous publications; Q3: Indicate time period used for identifying patients; Q4: Indicate whether or not subjects were consecutive if not population-based; Q5: Indicate if evaluators of subjective components of study were masked to other aspects of status of the participants; Q6: Describe any assessments undertaken for quality assurance purpose; Q7: Explain any patient exclusions from analysis; Q8: Describe how confounding was assessed and/or controlled; Q9: If applicable, explain how missing data were handled in the analysis; Q10: Summarize patient response rates and completeness of data collection; Q11: Clarify what follow-up, if any, was expected and the percentage of patients for which incomplete data or follow-up was obtained.

**Table S8.** Quality scores for assessing the risk of bias of in the included articles for randomized controlled study.

| **Score**  **Study (Year)** | **Q1** | **Q2** | **Q3** | **Q4** | **Total score** |
| --- | --- | --- | --- | --- | --- |
| Xie Y (2016)[24] | 2 | 1 | 1 | 1 | 5 |
| Wei X, et al. (2017)[31] | 2 | 2 | 1 | 2 | 7 |
| Wei X, et al. (2019)[35] | 2 | 2 | 1 | 2 | 7 |
| Li L, et al. (2017)[66] | 2 | 1 | 0 | 1 | 4 |
| Zhu J, et al. (2017)[68] | 2 | 0 | 1 | 1 | 4 |
| Ju JL, et al. (2019)[72] | 2 | 1 | 0 | 1 | 4 |

“No” was scored “0”, “unclear” was scored “1”, “Yes” was scored “2”. ≤3 scores: low quality; 4-5 scores: moderate quality; 6-7 scores: high quality. Q1: The generation of random sequences:1) adequate: random numbers or similar methods generated by computers or random number table (two points); 2) unclear: random test without random distribution method (one point); and 3) inadequate: alternate distribution method, such as odd and even numbers (zero point); Q2: Randomization: 1) adequate: center or pharmacy control allocation scheme, or containers with consistent sequence numbers, onsite computer control, sealed opaque envelopes, or other methods so that clinicians and participants cannot predict the allocation sequence (two points); 2) unclear: only using a random number table or other random allocation scheme (one point); 3) inadequate: alternate distribution, case number, and any other measures cannot prevent predictability packets; and 4) unused (zero point); Q3: Blindness: 1) adequate: using completely consistent placebo tablets or similar methods (two points); 2) unclear: just having the statement of blindness, but without description (one point); and 3) inadequate: not by double-blindness or way of blindness is not appropriate, such as the comparison of tablets and injections (zero point); and Q4: Follow-up: describing the number and reasons for withdrawal (one point), without the number or reasons for withdrawing (zero point).

**Appendix E**

**
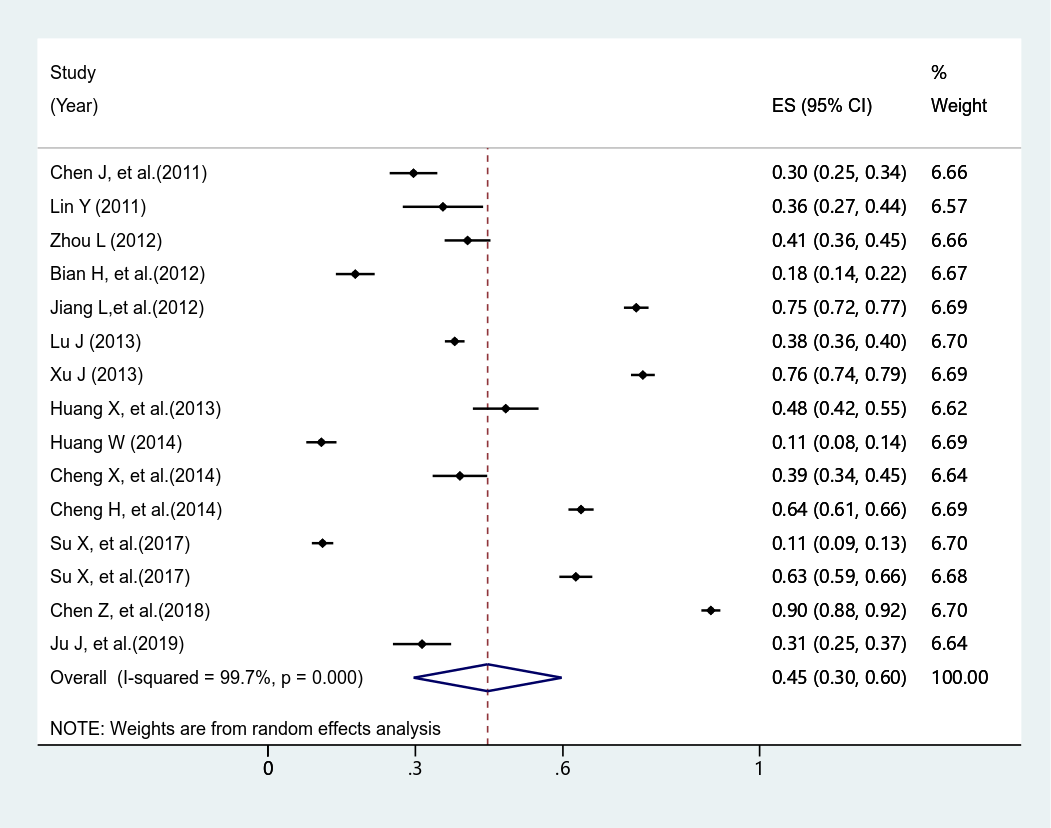
**

**Fig. S1.** Forest plot of the studies for pathogen detection of inpatient department.

**Table S9.** Forest plot of the studies for risk of antibiotic utilization of children and their caregivers lack of skills and knowledge

| **Question** | **Study (Year)** | **Number of**  **Right Responders** | **Number of Responders** | **Random-effects meta -analysis** |
| --- | --- | --- | --- | --- |
| antibiotics and anti-inflammatory drugs are the same drugs | Yu M, et al. (2014)[84] | 180 | 481 | 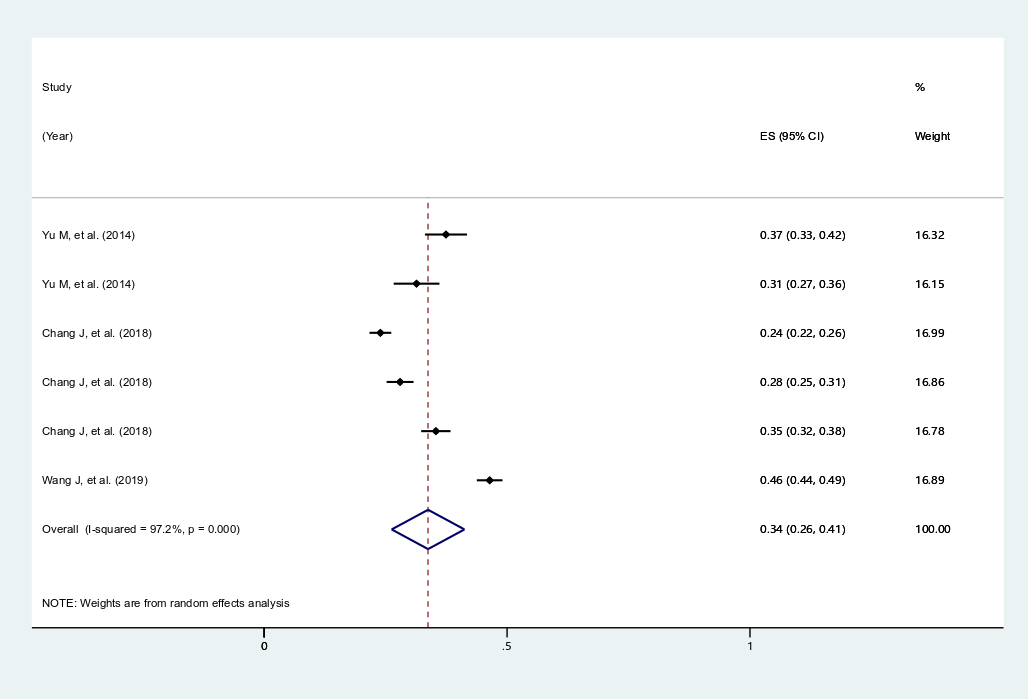 |
|  | Yu M, et al. (2014)[84] | 117 | 373 |  |
|  | Chang J, et al. (2018)[92] | 332 | 1388 |  |
|  | Chang J, et al. (2018)[92] | 282 | 1008 |  |
|  | Chang J, et al. (2018)[92] | 340 | 962 |  |
|  | Wang J, et al. (2019)[96] | 635 | 1368 |  |
| antibiotics can cure infections caused by virus | Cui F, et al. (2015)[77] | 629 | 1367 | 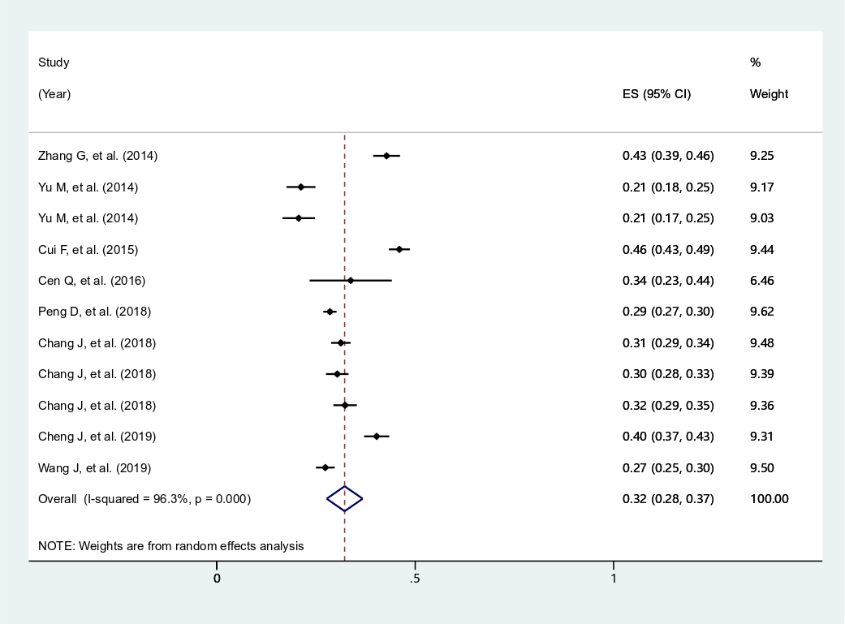 |
|  | Zhang G, et al. (2014)[83] | 350 | 818 |  |
|  | Yu M, et al. (2014)[84] | 102 | 481 |  |
|  | Yu M, et al. (2014)[84] | 77 | 373 |  |
|  | Cen Q, et al. (2016)[86] | 27 | 80 |  |
|  | Peng D, et al. (2018)[80] | 800 | 2806 |  |
|  | Chang J, et al. (2018)[92] | 434 | 1388 |  |
|  | Chang J, et al. (2018)[92] | 306 | 1008 |  |
|  | Chang J, et al. (2018)[92] | 311 | 962 |  |
|  | Cheng J, et al. (2019) [95] | 371 | 921 |  |
|  | Wang J, et al. (2019)[96] | 374 | 1368 |  |
|  |  |  |  |  |
| antibiotics can be used to treat common cold | Cui F, et al. (2015)[77] | 468 | 1402 | 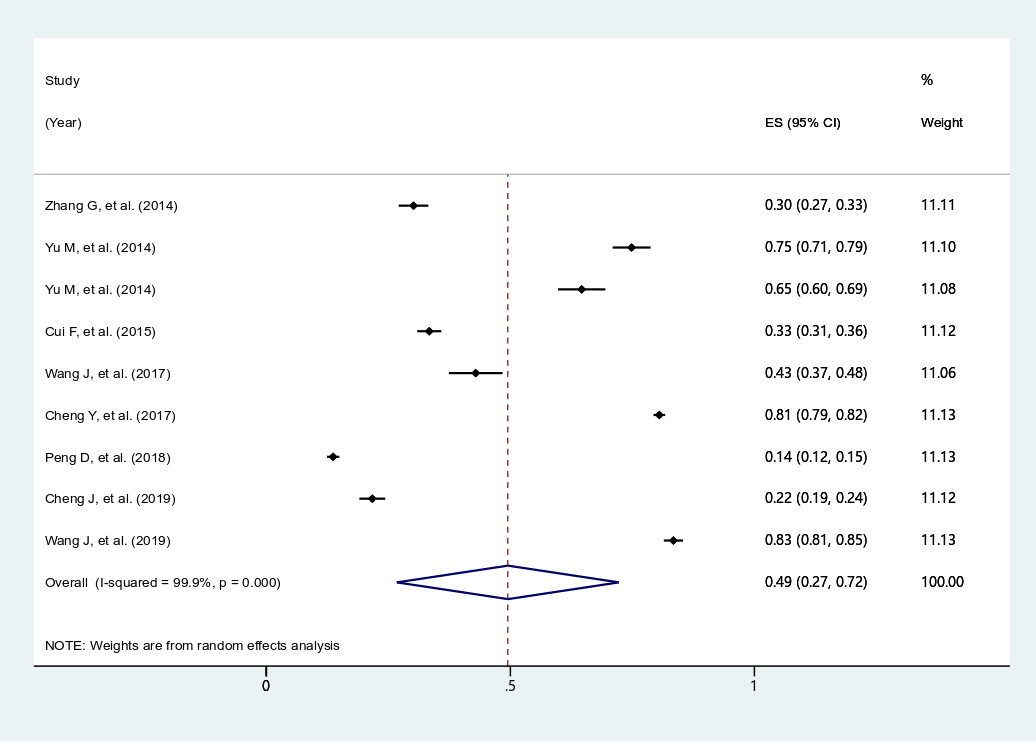 |
|  | Zhang G, et al. (2014)[83] | 263 | 872 |  |
|  | Yu M, et al. (2014)[84] | 360 | 481 |  |
|  | Yu M, et al. (2014)[84] | 241 | 373 |  |
|  | Wang J, et al. (2017)[89] | 133 | 310 |  |
|  | Cheng Y, et al. (2017)[91] | 3391 | 4211 |  |
|  | Peng D, et al. (2018)[80] | 384 | 2806 |  |
|  | Cheng J, et al. (2019)[95] | 200 | 921 |  |
|  | Wang J, et al. (2019)[89] | 1141 | 1368 |  |
|  |  |  |  |  |
| antibiotics can be used to treat pharyngitis or nonsuppurative tonsillitis | Cui F, et al. (2015)[77] | 446 | 1403 | 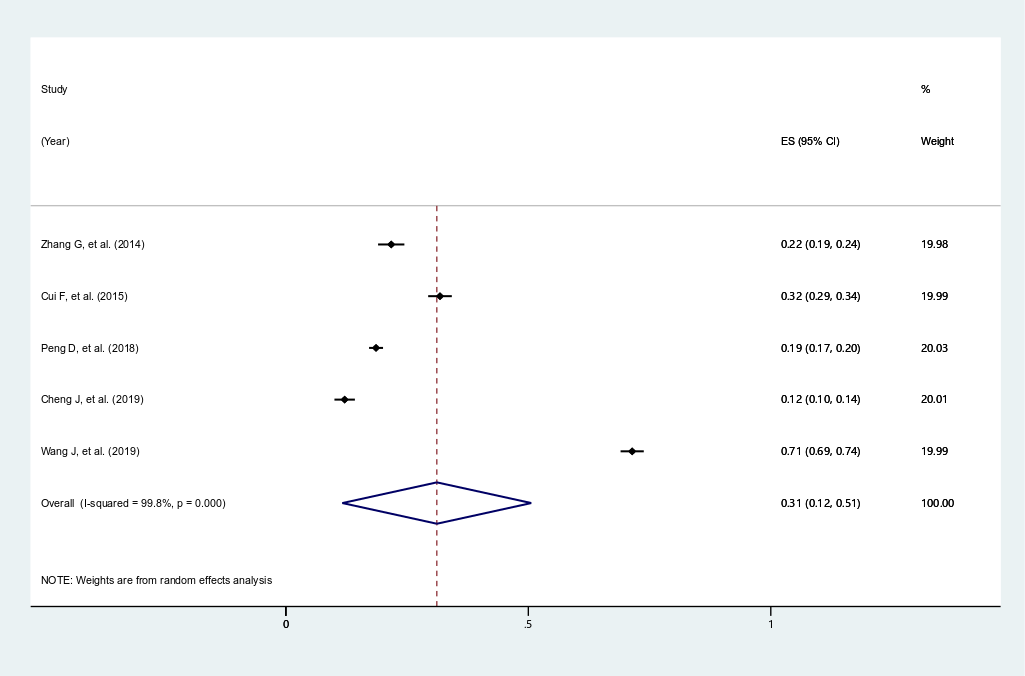 |
|  | Zhang G, et al. (2014)[83] | 193 | 888 |  |
|  | Peng D, et al. (2018)[80] | 522 | 2806 |  |
|  | Cheng J, et al. (2019)[95] | 112 | 923 |  |
|  | Wang J, et al. (2019)[96] | 977 | 1368 |  |
|  |  |  |  |  |
| antibiotics should only be obtained with a doctor’s prescription | Miao R, et al. (2020)[82] | 251 | 509 | 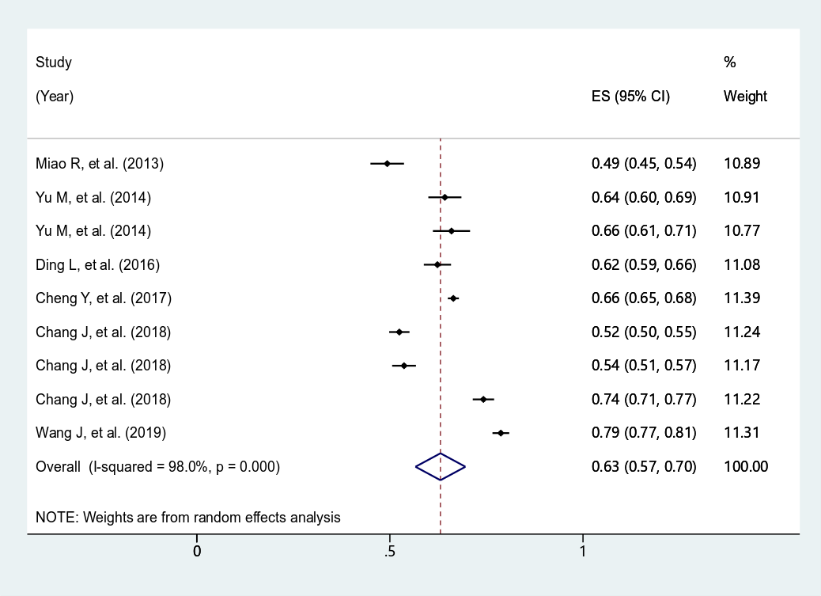 |
|  | Yu M, et al. (2014)[84] | 309 | 481 |  |
|  | Yu M, et al. (2014)[84] | 246 | 373 |  |
|  | Ding L, et al. (2016)[85] | 453 | 727 |  |
|  | Cheng Y, et al. (2017)[91] | 2791 | 4201 |  |
|  | Chang J, et al. (2018)[92] | 728 | 1388 |  |
|  | Chang J, et al. (2018)[92] | 541 | 1008 |  |
|  | Chang J, et al. (2018)[92] | 714 | 962 |  |
|  | Wang J, et al. (2019)[96] | 1077 | 1368 |  |
|  |  |  |  |  |
| inappropriate use of antibiotics can reduce the effectiveness of antibiotics | Cui F, et al. (2015)[77] | 885 | 1414 | 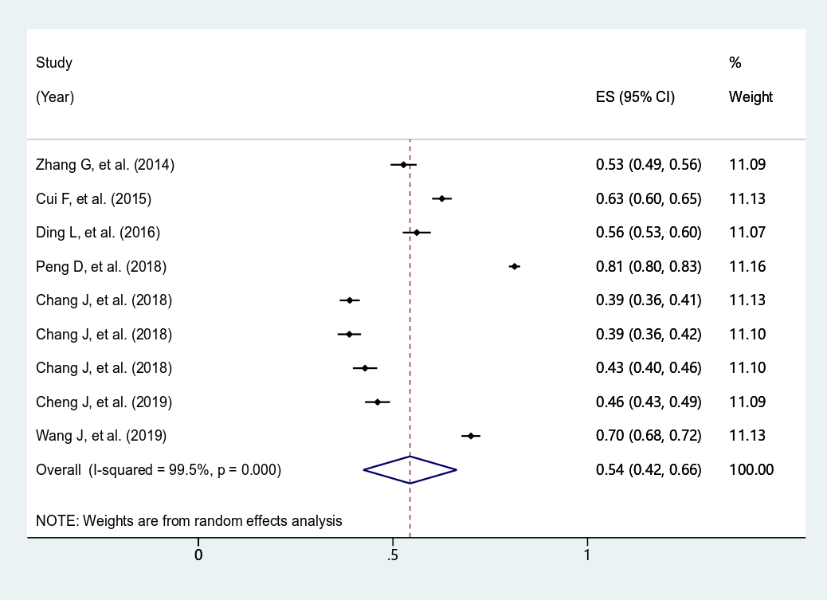 |
|  | Zhang G, et al. (2014)[83] | 453 | 859 |  |
|  | Ding L, et al. (2016)[85] | 408 | 727 |  |
|  | Peng D, et al. (2018)[80] | 2280 | 2806 |  |
|  | Chang J, et al. (2018)[92] | 540 | 1388 |  |
|  | Chang J, et al. (2018)[92] | 391 | 1008 |  |
|  | Chang J, et al. (2018)[92] | 412 | 962 |  |
|  | Cheng J, et al. (2019)[95] | 424 | 921 |  |
|  | Wang J, et al. (2019)[96] | 958 | 1368 |  |
|  |  |  |  |  |

**References**

1. Moher D, Liberati A, Tetzlaff J *et al*: Preferred reporting items for systematic reviews and meta-analyses: the PRISMA statement. *Plos Medicine* 2009, 6(7):e1000097.

2. Liang X, Zhuo Y: Analysis of appliance of antibiotics about outpatient transfusion prescriptions in our hospital. *Guangzhou Medical Journal* 2010, 41(4):65-66.

3. Hu X: Analysis of the utilization of antibiotics in pediatric department of our hospital. *China Pharmacy* 2010, 21(44):4186-4188.

4. Chen C, Xin L, Tan F: Analysis on the use of antibacterials in pediatric outpatient department of our hospital. *China Practical Medicine* 2010, 5(29):9-10.

5. Zhang J: Analysis on antibiotic treatment among pediatric outpatients in our hospital in 2010. *Chinese Journal of Medicinal Guide* 2011, 13(12):2166, 2172.

6. Wang J, Li G, Xiang F: Analysis of antibiotic application in outptient pediatric presccriptions. *Modern Hospitals* 2011, 11(5):57-58.

7. Li X: The application situation analysis of antibiotic usage in outpatient department with acute upper respiratory infections in our hospital. *China Modern Medicine* 2011, 18(9):150-151.

8. Liang J, Liang Y: Clinical observation of 500 cases with antibiotics in outpatient department of pediatrics. *Contemporary Medicine* 2012(30):144-145.

9. Huang X, Xie J, Xu Y *et al*: The investigation research of the prescription of antibiotic drugs in outpatient of the pediatric department of one hospital. *Chinese Journal of Clinical Rational Drug Use* 2012, 5(36):1-2.

10. Li H: Analysis and evaluation of drug application in pediatric outpatients of our hospital. *China Medical Herald* 2012, 9(08):114-115.

11. Chen L: Our hospital antimicrobial prescription pediatric clinic is not reasonable use analysis. *Journal of North Pharmacy* 2012, 9(11):66-67.

12. Zhou H, Lin Z, Lv D *et al*: Investigation of the use of antibacterial drugs in pediatric outpatient department of our hospital. *Chinese Journal of Pharmacovigilance* 2012, 9(09):566-568.

13. Li L, Feng L: Analysis of drugs application for upper respiratory tract infection in pediatric outpatient. *China Medical Herald* 2013, 10(02):114-116.

14. Xiao B: Analysis of the use of antibiotics in 1553 outpatient pediatric patients. *Medical Journal of National Defending Forces in Southwest China* 2013, 23(07):779-780.

15. Chen H, Zhu F, Lu H: Application analysis of antibacterial drugs in pediatric outpatient department of Lianzhou People's Hospital. *China Medicine and Pharmacy* 2013, 3(11):64-65.

16. Liu H: The analysis of the pediatric outpatient anti-infective drug using. *Hebei Medicine* 2013, 19(03):436-439.

17. Miao C, Lei H, Rong Y *et al*: Medication analysis on pediatrics outpatient prescription at our hospital. *China Health Industry* 2014(4):5, 7.

18. Zeng Y: Analysis on the pediatric prescriptions in the outpatient. *The Medical Forum* 2014(1):121-123.

19. Gui M, Wang J: To analyze the service condition of antibacterial agents of upper respiratory tract infection prescription in pediatric outpatient department. *Chinese Community Doctors* 2014(15):5, 7.

20. Yuan Y, Cao L, Yu X *et al*: Prescriptions of antibiotics for children with upper respiratory infections in outpatient department. *Chinese Journal of General Practitioners* 2015, 14(8):616-620.

21. Xu P, Lin R: Investigation on current situation of using antibiotics for acute upper respiratory infection in Children. *Journal of Pediatric Pharmacy* 2015, 21(7):47-49.

22. Xu X, Liu T: Analysis of usage of anti-infection drugs in department of pediatrics in our hospital. *China Medicine and Pharmacy* 2015, 5(24):72-74.

23. Gao Y: Analysis of antibiotics in pediatric patients with acute upper respiratory tract infection. *Chinese Community Doctors* 2016, 32(19):13-14.

24. Xie Y: MTP rationality of prescription and the effect of interventions in the treatment of acute upper respiratory tract infection in outpatient pediatrics. *Anhui Medical and Pharmaceutical Journal* 2016, 20(9):1784-1786, 1787.

25. Lin Y, Nie C, Yang L *et al*: Analysis and evaluation of prescriptions for pediatric upper respiratory tract infection. *China Licensed Pharmacist* 2016, 13(2):15-18.

26. Wang J: Evaluation of the use of antimicrobial drugs in pediatric outpatients with acute respiratory infection. *Journal of Tropical Medicine* 2016, 16(06):796-798.

27. Wang S: Analysis of antibacterials usage in respiratory tract infection of outpatient department of pediatrics in our hospital. *Chinese Journal of Drug Evaluation* 2017, 34(4):290-293.

28. Pan Y: Analysis on application of oral medicine for upper respiratory tract infections in our hospital. *Chinese and Foreign Medical Research* 2017, 15(13):142-143.

29. Nie C, Zhang J, Zhao Q *et al*: Investigation and analysis of antibiotics application in pediatric clinic of our hospital. *Modern Hospital* 2017, 17(08):1223-1225.

30. Zhang Z, Hu Y, Zou G *et al*: Antibiotic prescribing for upper respiratory infections among children in rural China: a cross-sectional study of outpatient prescriptions. *Global Health Action* 2017, 10(1):1287334.

31. Wei X, Zhang Z, Walley JD *et al*: Effect of a training and educational intervention for physicians and caregivers on antibiotic prescribing for upper respiratory tract infections in children at primary care facilities in rural China: a cluster-randomised controlled trial. *Lancet Global Health* 2017, 5(12):e1258-e1267.

32. Wei X, Yin J, Walley JD *et al*: Impact of China's essential medicines scheme and zero-mark-up policy on antibiotic prescriptions in county hospitals: a mixed methods study. *Tropical medicine & international health : TM & IH* 2017, 22(9):1166-1174.

33. Wang Z, Li L, Huang F *et al*: Analysis of rational drug use of the pediatric prescriptions from 45 grassroots hospitals in an area. *Pharmaceutical Administration* 2018, 27(17):86-89.

34. Wang C: Analysis of the use of antibiotics in outpatients with acute upper respiratory tract infections in pediatrics department. *Clinical Research and Practice* 2019, 4(03):118-119.

35. Wei X, Zhang Z, Hicks JP *et al*: Long-term outcomes of an educational intervention to reduce antibiotic prescribing for childhood upper respiratory tract infections in rural China: Follow-up of a cluster-randomised controlled trial. *Plos Medicine* 2019, 16(2):e1002733.

36. Peng L, Wang Z, Zhang Y: Analysis of rational use of antibiotic drugs in hospital pediatrics based on children's drug utilization index and drug safety. *Boletin De Malariologia Y Salud Ambiental* 2019, 59(1):89-95.

37. Cui Y: Analysis of antibiotics in hospitalized children. *Chinese Journal of Clinical Rational Drug Use* 2011, 04(23):43-44.

38. Qiu Z, Xie L, Xie H: Application analysis on antibiotics in pediatric patient with acute upper respiratory tract infection. *Chinese Journal of Clinical Rational Drug Use* 2011, 04(25):4-5.

39. Chen J, Wen Z: Research study on antibiotic application for bronchopneumonia of the children in-patient in pediatrics in 2010. *International Medicine and Health Guidance News* 2011, 17(24):3075-3078.

40. Lin Y: Analysis of antibiotics application in pediatric upper respiratory tract infection. *Chinese Journal of Nosocomiology* 2011, 21(12):2576-2577.

41. Liang Z, Li L, Li K: Analysis of the use of antibiotic in pediatric inpatients. *Medical Recapitulate* 2011, 17(12):1890-1891.

42. Zhou L: The analysis of nosocomial infection and antibiotics usage in hospitalized neonates. *China Practical Medicine* 2012, 07(15):32-34.

43. Bian H, Zhang T: Investigation of drug use in pediatrics in our hospital. *Chinese Journal of Pharmacoepidemiology* 2012, 21(3):138-139, 145.

44. Fang J: Antibiotics application in 196 pediatric patients with acute upper respiratory infection. *Journal of Modern Clinical Medicine* 2012, 38(2):123-124.

45. Wu W: An empirical analysis of antibiotic drug usage in pediatrics. *World Health Digest Medical Periodieal* 2012(39):72-73.

46. He M: Investigation of antibiotic use in hospitalized children with respiratory tract infection in our hospital from 2009 to 2011. *Gems of Health* 2012(4):389.

47. Guan L: Investigation of antibiotic use in pediatric inpatient with acute upper respiratory tract infections. *World Health Digest Medical Periodieal* 2012, 9(49):231-232.

48. Jiang L, Yang P: Investigation and analysis of nosocomial infection and antibiotic application in 1148 hospitalized children. *Journal of Frontiers of Medicine* 2012(20):94-95.

49. Zhu J, Shen N, Wang Y *et al*: Application of antibiotics in neonatal wards. *Chinese Journal of Nosocomiology* 2012, 22(17):3836-3837, 3840.

50. Wang Q, Sun C, Xu Y: Analysis on the usage of antibacterials in pediatric inpatients. *Chinese Journal of Drug Application and Monitoring* 2012, 9(06):348-350.

51. Che Y, Dong J: Use of antibiotics for neonates in a basic hospital and influencing factors analysis. *Chinese Journal of Woman and Child Health Research* 2013, 24(3):296-298.

52. Lu J, Huang J: Clinical analysis of antibiotics application for children with acute respiratory infection in Qinzhou. *Journal of Clinical Pulmonary Medicine* 2013, 18(12):2210-2212.

53. Xu J: Analysis of the usage of antibacterials in pediatric inpatients. *Chinese Journal of Pharmacoepidemiology* 2013, 22(4):196-198.

54. Huang X, Zhang W, Xu Q: Investigation of 215 medical records in internal medicine of pediatrics and analysis of antimicrobial drug Use. *Journal of Pediatric Pharmacy* 2013, 19(3):38-42.

55. Zhan J, Chen Q: Investigation and analysis of the application condition of antibacterial agents on hospitalization children in our hospital. *Nei Mongol Journal of Traditional Chinese Medicine* 2013, 32(18):96-97.

56. Li C, Ren N, Wen X *et al*: Changes in antimicrobial use prevalence in China: results from five points prevalence studies. *Plos One* 2013, 8(12):e82785.

57. Yao Q, Luo Z: Clinical application of antibiotic in department of pediatrics. *Chinese Journal of Clinical Rational Drug Use* 2014(15):1-2.

58. Wu M, Chen Y, Wu X: Investigation of pediatric medical records and analysis of antimicrobial drug application in a certain hospital. *Journal of Navy Medicine* 2014(4):260-262.

59. Zhu G, Wu X: Antibiotics usage analysis in pediatric inpatients. *Journal of Modern Medicine & Health* 2014, 30(21):3239-3240.

60. Liu Y: Survey and analysis of the use of antibacterials in pediatric inpatients of our hospital. *China Licensed Pharmacist* 2014, 11(11):13-15.

61. Huang W: Analysis of the use of antibiotic in our hospital in 2013 were from January to December in pediatrics. *China Health Industry* 2014, 11(21):35-36.

62. Huang G, Miao D, Lai X *et al*: Investigation of use of antibiotics by hospitalized neonates. *Chinese Journal of Nosocomiology* 2014, 24(01):97-98.

63. Cheng X, Yan X, Lin S: The Investigation and analysis of antimicrobial drugs used by 258 pediatric inpatients. *Journal of Hubei University for Nationalities Medical Edition* 2014, 31(02):40-41.

64. Cheng H, Wu D: Survey of antimicrobial agents use in pediatric inpatients. *Chinese Journal of Pharmaco Epidemiology* 2014, 23(12):737-740.

65. Chen Y, Ye J: Pharmaceutical intervention on the use of antibiotics in the neonatology of X-hospital. *China Health Industry* 2015(16):12-14.

66. Li L, Xu H, Zhou J *et al*: Application effect of clinical pathway management in infantile capillary bronchitis and the impact on antibiotics utilization ratio. *Chinese Journal of Clinical Rational Drug Use* 2017, 10(12):35-36, 41.

67. Su X, Zhu H, Yuan H: Analysis of antibiotic use for bronchial pneumonia in pediatrics. *Journal of Kunming Medical University* 2017, 38(7):126-129.

68. Zhu J, Luo M, Li L *et al*: Promotion of PDCA cycle on rational usage of antibiotics in neonatal department. *Journal of Guangdong Pharmaceutical University* 2017, 33(5):649-653.

69. Li R, Zhang J, Li G: Analysis of clinical antibiotic use and adverse reactions in pediatrics. *Chinese Nursing Research* 2018, 32(23):3815-3817.

70. Chen Z, Xiao Y: Analysis on usage of antibacterial drugs in inpatient children of Tianjin Children's Hosptial in 2016. *Drugs & Clinic* 2018, 33(03):672-675.

71. Zhang JS, Liu G, Zhang WS *et al*: Antibiotic usage in Chinese children: a point prevalence survey. *World Journal of Pediatrics: WJP* 2018, 14(4):335-343.

72. Ju J: Study on the effect of pharmacist intervention on rational use of pediatric antibiotics. *China & Foreign Medical Treatment* 2019, 38(7):127-129.

73. Wei W, Wang XF, Liu JP *et al*: Status of antibiotic use in hospitalized children with community-acquired pneumonia in multiple regions of China. *Chinese Journal of Contemporary Pediatrics* 2019, 21(1):11-17.

74. Niu J: Analysis on the treatment and intervention of pediatric upper respiratory tract infection in hospital. *Chinese Community Doctors* 2020, 36(16):18.

75. Miao R, Wan C, Wang Z *et al*: Inappropriate antibiotic prescriptions among pediatric inpatients in different type hospitals. *Medicine (Baltimore)* 2020, 99(2):e18714.

76. Zhang M, Ma XY, Feng ZQ *et al*: Survey of antibiotic use among hospitalised children in a hospital in Northeast China over a 4-year period. *Journal for specialists in pediatric nursing: JSPN* 2020, 25(2):e12282.

77. Cui F, Yuan Y, Cui H *et al*: A survey on the knowledge of antibiotics of children parents in three children-hospitals of Beijing. *Chinese Journal of Medicine* 2015(8):67-70.

78. Zhao M, Nan W, Jiao X *et al*: Survey on the parent situation of antibacteriol drugs use for children. *Northwest Pharmaceutical Journal* 2016, 31(02):200-202.

79. Yin Y, Cui N: Antibiotics use among preschool children and parental cognition in Ji'nan city. *Chinese Journal of Public Health* 2018, 34(1):118-120.

80. Peng D, Zhou X: Parents' antibiotic use for children in Ningbo: knowledge, behaviors and influencing factors. *Journal of Zhejiang University* 2018, 47(2):156-162.

81. Yao Z, Zhou J, Li Y *et al*: Prevalence of self-medication with antibiotics in kindergarten children of Guangzhou city. *Chinese Journal of Public Health* 2013, 29(10):1485-1487.

82. Miao R: Investigation on the impact of parents' cognitive level of antibiotics on self-directed use of antibiotics in pupils. *Practical Preventive Medicine* 2013, 20(1):42-45.

83. Zhang G, Cao L, Yuan Y: 10 years' changes on parent's antibiotic knowledge in Beijing. *Chinese Journal of Medicine* 2014(10):41-44.

84. Yu M, Zhao G, Stålsby LC *et al*: Knowledge, attitudes, and practices of parents in rural China on the use of antibiotics in children: a cross-sectional study. *Bmc Infectious Diseases* 2014, 14:112.

85. Ding L, Sun W, Li Y *et al*: Studying on the status of rural parents' cognition on antibiotics and its influencing factors. *Chinese Health Service Management* 2016, 33(2):111-114.

86. Cen Q, Dai D: The relationship between novice mother and neonates in use of antibiotic cold medications and the effects of health education. *Drug Evaluation* 2016, 13(21):47-50.

87. Li R, Xiao F, Zheng X *et al*: Antibiotic misuse among children with diarrhea in China: results from a national survey. *Peerj* 2016, 4:e2668.

88. Zhang Z, Zhan X, Zhou H *et al*: Antibiotic prescribing of village doctors for children under 15 years with upper respiratory tract infections in rural China: A qualitative study. *Medicine (Baltimore)* 2016, 95(23):e3803.

89. Wang J, Huang C, Li Z *et al*: Knowledge and behavior of antibiotic use for upper respiratory tract infection among parents of young children in Changsha city. *Chinese Journal of Public Health* 2017, 33(3):415-418.

90. Zhang Y, Lin S, Li G *et al*: Cognition and usage of antibiotics in caregivers of 0-6 years old children in the rural area of Weinan City, 2015. *Practical Preventive Medicine* 2017, 24(2):196-198.

91. Cheng YC, Pan YP, Zhang Y *et al*: Investigation of the cognition and behavior on drug safety in Beijing middle school students. *Beijing Da Xue Xue Bao Yi Xue Ban* 2017, 49(6):1038-1043.

92. Chang J, Lv B, Zhu S *et al*: Non-prescription use of antibiotics among children in urban China: a cross-sectional survey of knowledge, attitudes, and practices. *Expert Rev Anti Infect Ther* 2018, 16(2):163-172.

93. Fan W, Mu J, Song X *et al*: Investigation on antibiotic cognition and use of parents of preschool children in Tianjin. *Chinese Primary Health Care* 2019, 33(4):61-62.

94. Ge Y, Chipenda DS, Liao XP: Advanced neonatal medicine in China: Is newborn ward capacity associated with inpatient antibiotic usage? *Plos One* 2019, 14(8):e219630.

95. Cheng J, Chai J, Sun Y *et al*: Antibiotics use for upper respiratory tract infections among children in rural Anhui: children's presentations, caregivers' management, and implications for public health policy. *Journal of Public Health Policy* 2019, 40(2):236-252.

96. Wang J, Sheng Y, Ni J *et al*: Shanghai parents' perception and attitude towards the use of antibiotics on children: A cross-sectional study. *Infection and Drug Resistance* 2019, 12:3259-3267.

97. Ye D, Yan K, Zhang H *et al*: A survey of knowledge, attitudes and practices concerning antibiotic prescription for upper respiratory tract infections among pediatricians in 2018 in Shaanxi Province, China. *Expert Review of Anti-Infective Therapy* 2020, 18(9):927-936.

98. Wei X, Deng S, Haldane V *et al*: Understanding factors influencing antibiotic prescribing behaviour in rural China: a qualitative process evaluation of a cluster randomized controlled trial. *Journal of Health Services Research & Policy* 2020, 25(2):94-103.

99. Zhang J, Cameron D, Quak SH *et al*: Rates and determinants of antibiotics and probiotics prescription to children in Asia-Pacific countries. *Beneficial Microbes* 2020, 11(4):329-338.
